# Flavonoids, Chalcones, and Their Fluorinated Derivatives—Recent Advances in Synthesis and Potential Medical Applications

**DOI:** 10.3390/molecules30112395

**Published:** 2025-05-30

**Authors:** Jakub Kubiak, Piotr Szyk, Beata Czarczynska-Goslinska, Tomasz Goslinski

**Affiliations:** 1 Chair and Department of Chemical Technology of Drugs, Poznan University of Medical Sciences, Rokietnicka 3, 60-806 Poznan, Poland; 86216@student.ump.edu.pl (J.K.);; 2 Doctoral School, Poznan University of Medical Sciences, Bukowska 70, 60-812 Poznan, Poland; 3 Chair and Department of Pharmaceutical Technology, Poznan University of Medical Sciences, Rokietnicka 3, 60-806 Poznan, Poland; bgoslinska@ump.edu.pl

**Keywords:** antibacterial agents, anticancer agents, chalcones, flavonoids, fluorinated drugs

## Abstract

Flavonoids and chalcones, widely recognised for their diverse biological activities, have garnered attention due to their potential therapeutic applications. This review discusses fluorinated flavonoids and chalcones, focusing on their prospective anti-inflammatory, antidiabetic, anticancer, antiosteoporotic, cardioprotective, neuroprotective, hepatoprotective, antimicrobial, and antiparasitic applications. The enhanced biological activities of fluorinated derivatives, particularly the antibacterial, antiviral, and anticancer properties, are attributed to the introduction of fluorine groups, which increase lipophilicity and metabolic stability. Key findings indicate that fluorinated flavonoids and chalcones exhibit synergistic effects with antibiotics, inhibit bacterial efflux pumps, and reveal potent antiviral and anticancer properties. However, challenges such as cytotoxicity and structural optimisation have to be addressed. The synthesis of fluorinated flavonoids and chalcones is discussed, with emphasis on various synthetic methods such as condensation and cyclisation reactions starting from fluorinated precursors, as well as fluorination strategies, including the use of molecular fluorine or fluorinating agents. Fluorinated flavonoids and chalcones represent candidates for therapeutic development and have the potential to overcome drug resistance. However, further studies are necessary to adjust their pharmacological profiles.

## 1. Introduction

Flavonoids constitute a class of natural compounds and plant secondary metabolites possessing variable phenolic structures. These yellow dyes of most colourful plant organs [1,2] are present in vegetables, fruits, grains, bark, roots, stems, nuts, flowers, tea, coffee, and wine [3,4,5,6].

**Figure 1:**
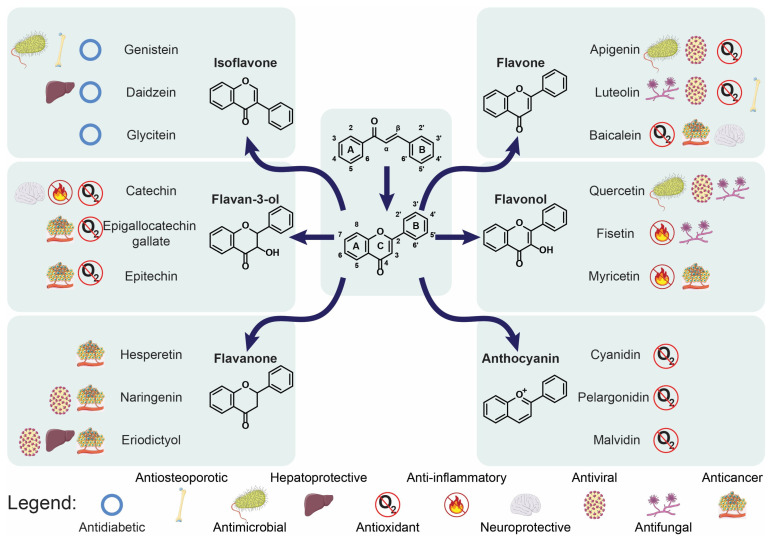
The structural representations of chalcone and flavonoid, with six major classes of flavonoids, as well as the crucial directions of their biological activity.

The chemical structures of almost all flavonoids are based on a 15-carbon flavone skeleton, C6-C3-C6, containing two benzene rings, A and B, linked by a heterocycle pyrone ring (C) that contains oxygen [7,8,9]. The position of the catechol ring (B-ring) attached to benzene (C-ring) differentiates flavonoid (2-phenylchromans) from isoflavonoid (3-phenylchromans) [9,10] and has an impact on the antioxidant capacity. The structural representations of chalcone and flavonoid are presented in Figure 1. Despite their wide structural diversity, flavonoids share a common feature, which is the presence of one (simple phenolic) or more (polyphenol) hydroxyl substituents, connected with one or more aromatic or benzene rings. The phenolic compounds may be grouped into six major classes: flavan-3-ols, flavones, flavonols, flavanones, isoflavones, and anthocyanins [7]. Moreover, chalcones, one of the major secondary metabolites of plants, are included in the flavonoid family [11].

Flavonoids are widely applied as preservatives, pigments, and antioxidants in the food, cosmetic, and pharmaceutical industries due to their excellent antioxidant properties, natural origin, low toxicity, and reasonable price [7]. The antioxidant action of flavonoids consists of various mechanisms. They are able to stimulate and protect antioxidant defences, prevent oxidation by directly scavenging reactive oxygen species (ROS), retarding ROS formation through the chelation of metal ions and the inhibition of the enzymes participating in the generation of free radicals [7,10,12]. Some flavonoids exhibit antifungal activity [13] or display photochemical properties used in beverages against light-induced colour deterioration [7,14]. Although flavonoids possess multiple favourable biological properties, their clinical use is limited by their low bioavailability and bioactivity resulting from their poor aqueous solubility, intensive metabolism, and low systemic absorption. Therefore, the development of novel delivery systems emerges as an approach for overcoming these drawbacks and broadening the application of dietary flavonoids [15].

## 2. Flavonoids and Chalcones—Biological Role and Medical Applications

The popularity of flavonoids results from their excellent antioxidant properties and contributes to their beneficial health activities, including anti-inflammatory, anticancer, antidiabetic, antibacterial, antiviral, antifungal, and antimalarial properties. They are also considered to exert neuroprotective and cardioprotective effects [16].

### 2.1. Anti-Inflammatory Activity

**Figure 2:**
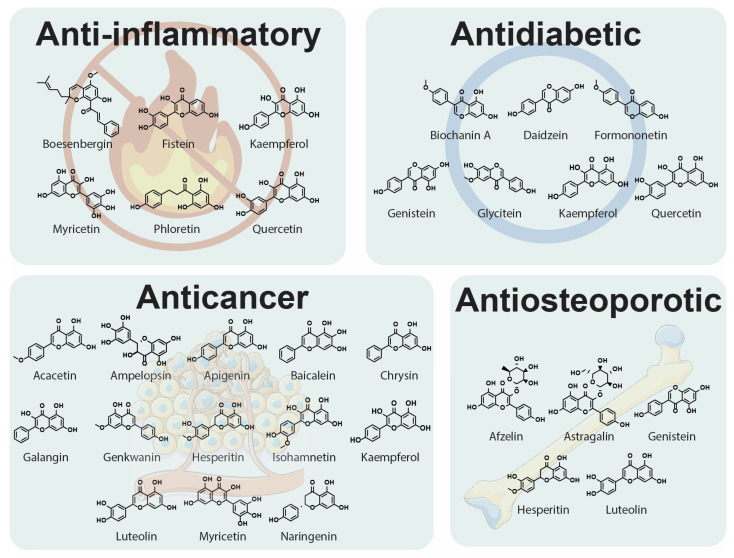
Representative chemical structures of flavonoids and chalcones with anti-inflammatory, antidiabetic, anticancer, and antiosteoporotic properties.

Flavonoids exert anti-inflammatory activities (Figure 2) through different mechanisms. They are able to inhibit the synthesis and actions of various pro-inflammatory mediators (eicosanoids, cytokines, adhesion molecules, and C-reactive protein), transcription factors, and regulatory enzymes involved in inflammation and its progression [17,18,19,20]. Hsieh et al. [20] analysed data obtained from the Nutrition and Health Survey in Taiwan 2005–2008 and found that C-reactive protein (CRP) levels were inversely related to flavonol, flavan-3-ol, and total flavonoid intakes, suggesting their anti-inflammatory contribution. According to Silva [21], flavonoids present in honey also exert anti-inflammatory activity by inhibiting both enzymes such as cyclooxygenase, lipoxygenase, nitric oxide synthase (iNOS), and pro-inflammatory mediators such as nitric oxide, cytokines, and chemokines. Flavonoids affect various kinases by suppressing the transcription factor, i.e., nuclear factor kappa-B (NF-*κ*B). Its inactivation has been associated with anti-inflammation, which was confirmed by Park et al. [18]. They noted that fisetin, kaempferol, myricetin, quercetin, and rutin inhibited IgE or phorbol-12-myristate 13-acetate and calcium ionophore A23187 (PMACI)-mediated histamine release in RBL-2H3 cells. Moreover, these flavonoids also contributed to the inhibition of the rise in intracellular calcium ions. Fisetin, quercetin, and rutin reduced gene expression and production of such pro-inflammatory cytokines as tumour necrosis factor-*α* (TNF-*α*), interleukin (IL)-1*β*, IL-6, and IL-8 in PMACI-stimulated human mast cells. Myricetin decreased TNF-*α* and IL-6 but not IL-1*β* and IL-8. Fisetin, myricetin, and rutin inhibited the activation of NF-*κ*B [16]. Polymethoxychalcones reveal anti-inflammatory properties due to the production of a high amount of IL-1*β*-induced prostaglandin E2 (PGE2), prostaglandin inhibitors, inflammatory cytokines, and normal human epidermal keratinocyte inhibitors. Some pyran-fused chalcones and trichalcones can impede the NF-*κ*B signalling complement system [22].

The antioxidant potential of flavonoids is another example of the mechanisms of their anti-inflammatory activity. They can simultaneously scavenge ROS and reduce their formation by either inhibiting enzymes or chelating transition metals involved in the generation of free radicals [12]. Different chalcones also possess antioxidant properties. Compounds occurring in tomatoes, such as phloretin-3′,5′-di-C-glucoside, panduratin A, boesenbergin, or phloretin present in apples reveal such features [11].

### 2.2. Antidiabetic Effect

Flavonoids (Figure 2) alleviate the pathogenesis of diabetes and its complications through the regulation of glucose metabolism, hepatic enzyme activities, and lipid profile [23]. Antidiabetic properties consist of the effects on blood sugar transporters because they regulate carbohydrate digestion, stimulate insulin secretion, limit apoptosis, and enhance the proliferation of pancreatic *β*-cells. They also influence insulin signalling, glucose uptake, and adipose deposition. They contribute to the reduction in insulin resistance, inflammation and oxidative stress in muscles and the promotion of translocation of glucose transporter type 4 (GLUT4) [24,25,26].

Ali et al. [27] studied the multi-targeting antidiabetic potential of quercetin and kaempferol. Both flavonoids exhibited appropriate ADMET (absorption, distribution, metabolism, excretion, and toxicity) profiles and demonstrated higher binding affinities for CRP, interleukin-1, dipeptidyl peptidase-4, peroxisome proliferator-activated receptor gamma, protein tyrosine phosphatase, and sodium-glucose co-transporter-1 compared to metformin, an antidiabetic drug. Both quercetin and kaempferol inhibited *α*-amylase activity, markedly reduced blood glucose levels and improved lipid profiles. The tested compounds revealed higher binding affinities for multiple targets than metformin. Ahmed et al. [28] assessed the antidiabetic effects of the isoflavonoids biochanin A, genistein, daidzein, glycitein, and formononetin on metabolic disorders and long-term complications induced by diabetes mellitus type 2. Genistein appeared to be a promising candidate for the prevention and treatment of diabetes. Biochanin A turned out to inhibit aldose reductase and acted through peroxisome proliferator-activated receptors (PPARs) *α*- and *γ*-modulation pathways that regulate glycaemic balance. The authors noted that isoflavonoids could activate gene expression through the stimulation of PPARs (*α*, *γ*), modulate carbohydrate metabolism, regulate hyperglycaemia, induce dyslipidaemia, reduce insulin resistance, impair oxidative stress, and modify adipocyte differentiation and tissue metabolism. Kaushal and Kaur [29] concluded that chalcones, thanks to their structure, can be chemically modified to enhance their interaction with enzymes involved in diabetes. They are able to inhibit *α*-glucosidase, *α*-amylase, aldose reductase, and tyrosine phosphatase, which are associated with the development of diabetes. Moreover, vanadium ion complexes with chalcone ligands can increase their insulin mimetic activity and potential for the treatment of hyperglycaemic conditions in the body. Proenca et al. [30] studied and confirmed *α*-glucosidase inhibition by flavonoids, as this mechanism is considered the most effective in reducing post-prandial hyperglycaemia from all available antidiabetic drugs used in the management of type 2 diabetes mellitus.

### 2.3. Anticancer Potential

Many research studies have indicated the relation between flavonoid consumption and its impact on the prevention and progression of breast, colon, liver, and lung cancer (Figure 2) [31]. Their therapeutic properties are based on angiogenesis modulation, anti-inflammatory potential, counteracting oxidative stress, cell cycle arrest, and apoptosis induction. All these mechanisms are mediated through the regulation of cellular signalling pathways such as phosphoinositide-3 kinase/RAC-alpha serine/threonine-protein kinase (PI3K/Akt), Wnt/*β*-catenin, and mitogen-activated protein kinase (MAPK) [31,32,33]. Flavonoids inhibit cancer growth and differentiation by their impact on such signalling pathways as signal transducers and activators of transcription (STAT5 and STAT3) and Janus kinase 2. These paths are important in cell growth, survival, and inflammatory responses [31,34]. The anticancer mechanism of flavonoids may also be related to the regulation of the competing endogenous RNA network. According to Li et al. [35], flavonoids, such as galangin, baicalein, and chrysin, can change the expression level of mRNAs in tumours and induce the differential expression of microRNAs and long non-coding RNAs [35]. What is interesting, flavones can act both as an antioxidant and as a pro-oxidant due to their structure [36]. Luteolin exerts antioxidant activity by regulating redox stress, whereas its pro-oxidant properties are helpful to induce apoptosis in tumour cells due to ROS-mediated damage to nucleotides and proteins. The main pathways involved in this process include suppression of the NF-κB signalling and activation of the JNK (c-Jun N-terminal kinase) pathway that induces TNF-mediated cytotoxicity to cancer cells [37]. In cancer cells, they regulate myeloperoxidase (MPO), which increases the oxidative stress and leads to apoptosis or necrosis [36]. MPO is foremost presented as a molecule that favours tumour initiation and progression due to the action of MPO-derived oxidants that are able to affect DNA [38]. Some flavonoids, such as naringenin, hesperidin, ampelopsin, luteolin, and myricetin, inhibit the enzyme xanthine oxidase, which is involved in the purine catabolism pathway, indirectly associated with cancer, and able to generate cytotoxic ROS [39,40]. Zhang et al. [41] investigated anticancer properties of flavonoids from *Trichosanthes kirilowii*—isorhamnetin, isorhamnetin-3-glucoside, kaempferol, apigenin, acacetin, chrysin, 7,8-dihydroxyflavanone, and genkwanin. Three flavonoids (isorhamnetin—IH, genkwanin—GN, and acacetin—Aca) brought about cell cycle arrest at the G2/M phase and apoptotic and autophagic cell death in human breast cancer cells. The authors also suspected that the interruption of the PI3K/AKT/mTOR/p70S6K signalling pathway contributed to the flavonoid-induced cell cycle arrest at the G2/M phase, apoptosis, and autophagy through reducing PI3K*γ*-p110 expression [41].

### 2.4. Antiosteoporotic Activity

Some flavonoids revealed potential bone-specific effects in several studies, including the enhancement of bone formation and the inhibition of bone resorption due to their impact on cell signalling pathways that modulate osteoblast and osteoclast differentiation (Figure 2) [42]. The main metabolite of hesperidin, hesperetin-7-*O*-glucuronide, activates bone morphogenic proteins (BMPs), which enhances the expression of the major transcription factor Runx2, stimulating osteoblast differentiation from multipotent mesenchymal stem cells in bone marrow [43]. Flavonoids may prevent bone loss by reducing the effects of oxidative stress or chronic low-grade inflammation [44]. Ma et al. [45] evaluated the in vitro antiosteoporotic activity of a new biflavonoid, podocarnone, and five known flavonoids isolated from *Pittosporum podocarpum*. The new compound exerted a stimulatory effect on osteoblastic proliferation and alkaline phosphatase (ALP) activity, as well as an inhibitory effect on osteoclastic tartrate-resistant acid phosphatase (TRAP) activity. Genistein revealed antiosteoporotic properties due to its oestrogen-like structure and thus could be considered a promising bioactive agent for the prevention of post-menopausal bone loss [45,46]. Afzelin, astragalin, and luteolin also influenced activities on both osteoblasts and osteoclasts [45]. The antiosteoporotic potential of some flavonoids results from their role in the Wnt-*β*-catenin pathway that stimulates bone formation and inhibits osteoclast activation [47]. Xiao et al. [48] studied the association between flavonoid intake and bone mineral density (BMD), considering the role of composite dietary antioxidant index (CDAI) in their relationship, using data from the National Health and Nutrition Examination Survey (NHANES). They analysed flavonoid intake, femur BMD, and osteoporosis in 10,225 subjects from NHANES 2007–2010 and 2017–2018 and noted the inverse relationship between flavonoid intake and the risk of osteoporosis evaluated on the basis of higher femur BMD. Similar conclusions were drawn by Cui et al. [49], who conducted research on the relationship between CDAI and BMD in a group of 2994 children and adolescents from NHANES 2007–2010. They searched for the link between CDAI and total spine, femur neck, and total femur BMD. The dietary intake of multiple antioxidants was positively associated with BMD in children and adolescents.

### 2.5. Cardioprotective Effect

Flavonoids also exert beneficial actions on the vascular system. They block platelet aggregation and the oxidation of low-density lipoprotein (LDL), ameliorate endothelial dysfunction, reduce blood pressure, improve antioxidant defences, and alleviate inflammatory responses [49]. The cardiovascular system is vulnerable to the oxidative stress triggered by ROS. Flavonoids have been proven to effectively prevent damage caused by ROS and other free radicals in the human body. Their phenolic moieties are highly susceptible to oxidation [4,50]. The highly reactive hydroxyl groups of the flavonoids inactivate free radicals by the donation of a proton [51,52]. The antioxidant activity of these compounds brings about the diminution of the oxidative damage to cellular components [51]. Flavonoids can exert antihypertensive effects by interacting with ion channels, inhibiting angiotensin-converting enzymes, maintaining the action of the renin–angiotensin–aldosterone system, and increasing NO-mediated vasodilation [51,53]. Moreover, they are supposed to potentiate bradykinin effects, decrease endothelin levels, and exert antiapoptotic effects on cardiomyocytes during oxidative damage, which lowers the risk of myocardial infarctions. They prevent LDL from oxidation, limiting the progression of arteriosclerosis [51,54]. Flavonoids also exert antiplatelet aggregation effects by inhibiting the relevant enzymes and signalling pathways, contributing to the lower production of oxidants and better re-establishment of blood in the ischaemic zone. All these multifaceted activities make flavonoids suitable agents for alleviating ischaemia–reperfusion injury [53].

### 2.6. Neuroprotective Effects

**Figure 3:**
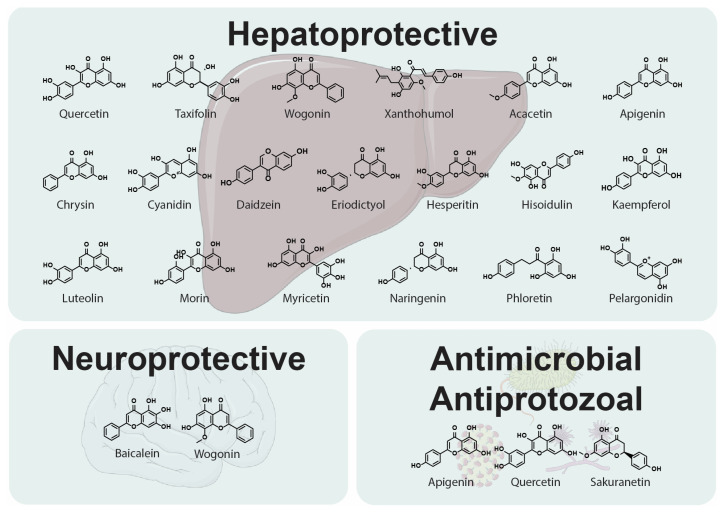
Representative chemical structures of flavonoids and chalcones with hepatoprotective, neuroprotective, as well as antimicrobial and antiprotozoal properties.

Flavonoids appear to be effective antioxidants and are useful for reducing the levels of oxidative stress and inflammation in neural cells. They can act as neuroprotectants (Figure 3) due to their ability to enhance the glyoxalase pathway, which accounts for the detoxication of reactive dicarbonyl compounds, including methylglyoxal (MG). This property is of great importance as the accumulation of MG is often associated with Alzheimer’s disease, Parkinson’s disease, ageing, and autism spectrum disorder [55]. Pan et al. [56] studied the utility of baicalein, baicalin, and wogonin, extracted from *Scutellaria baicalensis Georgi*, for the treatment of ischaemia-induced neurotoxicity and damage in the brain and retina. They explained that the neuroprotective effects were triggered by the flavonoids with their anti-inflammatory, antioxidative, and antiapoptotic capabilities. Scavenging excessive ROS restores homeostatic balance in oxidative stress conditions such as ischaemia, and it is a neuroprotective target in neurodegenerative disorders. Moreover, flavonoids inhibit the release of pro-inflammatory cytokines. Lastly, baicalein, baicalin, and wogonin have been reported to modulate the MAPK pathway and the production of apoptotic factors in ischaemia-injured brain and retina, which results in rescuing neurons in the brain and retina [56]. Yang et al. [57] investigated the therapeutic effect of baicalein from the root of *S. b. Georgi* administered in the subacute phase of cerebral ischaemia–reperfusion injury in a rat model. Baicalein reduced neurobehavioral deficits and decreased brain infarct volume. As a result of caspase-3 expression, neuronal apoptosis was reduced, and neuronal loss was alleviated. Baicalein promoted the phosphorylation of the PI3K/Akt/mTOR signalling pathway and probably suppressed autophagy [57]. Rowhanirad and Taherianfard [58] investigated the effects of chalcones from a Japanese herb *Angelica keiskei* Ashitaba (ChA) on cuprizone-induced demyelinating changes in the C57BL6 mice model of multiple sclerosis. The animals obtained normal diets (control group: CNT) or cuprizone-supplemented diets either without ChA (cuprizone group: CPZ) or with low or high (300, 600 mg/kg/day) doses of ChA (ChA-treated groups: CPZ + ChA300/600). The scientists noted that ChA co-treatment evidently decreased the degree of demyelination in the corpus callosum and the serum and brain levels of TNF-*α* in comparison to the control group. Moreover, after the application of the higher dose of ChA, a considerable improvement in behavioural responses and an increase in brain-derived neurotrophic factor (BDNF) levels in the serum and brain of the CPZ + ChA600 group were observed.

### 2.7. Hepatoprotective Activity

ROS are produced as a result of the metabolic processes of various compounds in the liver, causing damage to liver tissue. For this reason, antioxidants are necessary to inhibit free radicals and maintain the oxidant and antioxidant balance in the liver. They also prevent the generation of lipoxygenase and cyclooxygenase enzymes, which are able to co-oxidise molecules. Oxidative agents and lipid peroxidation products release inflammatory mediators such as fibrogenic growth factors, prostaglandins, and cytokines. Flavonoids (Figure 3) are considered a first-line free radical scavenger among all types of natural antioxidants [59].

Kuttiappan et al. [60] evaluated the cytoprotective and hepatoprotective properties of flavonoids present in an ethanolic extract from the *Sesbania grandiflora* plant. The cytotoxicity and antioxidant activity were studied on hepatocellular carcinoma G2 (HepG2) cell lines. Serum glutamic oxaloacetic transaminase, serum glutamic pyruvic transaminase, triglyceride, bilirubin, and total protein levels were measured to assess hepatotoxicity in animal models, and their reductions confirmed the hepatoprotective potential of the extract. Conversely, elevated levels of hepatic injury markers in the untreated group indicated liver damage. Flavonoid treatment reduced cell viability but did not affect antioxidant parameters in hepatocytes. Moreover, the damaged hepatocyte architecture was restored. Molecular docking studies revealed the binding affinities of flavonoids for peroxisome proliferator-activated receptor alpha PPAR*α*.

Kim et al. [61] analysed the effects of flavonoids on liver damage caused by benzo[a]pyrene (B[a]P), which could lead to the onset and progression of various diseases, including cancer. B[a]P generates reactive oxygen species within cells, and flavonoids can be used to counteract its effects. They create defence mechanisms against intracellular ROS by scavenging free radicals and regulating the sophisticated system of antioxidant enzymes. The researchers investigated the superoxide anion-scavenging potency and antioxidation capacity of quercetin, rutin, morin, acacetin, hispidulin, hesperidin, and naringin. They concluded that different flavonoids demonstrate antioxidant effects via various mechanisms. Rutin appeared as the strongest scavenger, followed by quercetin, in contrast to naringin and hesperidin, revealing no antioxidative effects [61]. Several studies have shown the regulatory role of flavonoids in modulating nuclear factor erythroid 2-related factor 2, a transcription factor modulating defence against oxidative damage through the expression of appropriate genes. This activity leads to a reduction in ROS in the liver [62]. Flavonoids such as naringenin, hesperidin, eriodictyol, taxifolin, apigenin, wogonin, luteolin, chrysin, daidzein, pelargonidin, cyanidin, phloretin, xanthohumol, quercetin, kaempferol, rutin, and myricetin efficiently diminish ROS and malondialdehyde, which is a marker reflecting oxidative stress levels. Flavonoids evidently have the potential to ameliorate the hepatotoxic effects induced by B[a]P or counteract the deleterious impact of ROS-induced liver damage [61].

### 2.8. Antimicrobial and Antiprotozoal Potential

Several natural compounds have demonstrated antibacterial, antiviral, or antifungal effects [63]. Polyphenolic compounds such as flavonoids and chalcones (Figure 3) have been widely studied for their antibacterial property due to their tendency to inhibit the growth of a broad range of pathogenic microorganisms, including multidrug-resistant bacteria [64]. They can inhibit bacterial cell envelope synthesis, as reported by Zhang et al. [65]. They noted that quercetin, apigenin, and sakuranetin blocked *β*-hydroxyacyl-ACP dehydrase from *Helicobacter pylori* with IC_50_ values of 39.3 μM, 11.0 μM, and 2.0 μM, respectively. They also proposed that these flavonoids could act as competitive inhibitors of *β*-hydroxyacyl-acyl carrier protein dehydratase from *H. pylori* by competing with the substrate crotonyl-CoA [65]. Flavonoids, as significant topoisomerase inhibitors, can affect nucleic acid synthesis [64]. Flavonoids can also quickly stop bacterial movement by blocking swarm motility to prevent bacterial adhesion as well as colonisation, as bacterial movement and attachment occur at different times [66]. The antimicrobial mechanisms of flavonoids also include the inhibition of the electron transport chain and ATP synthesis, bacterial toxins, biofilm formation, and bacterial plasma membrane disruption [64,67]. Flavonoids can act as antiviral agents at different stages of DNA and RNA viral infection, such as viral attachment, entry, replication, and translation of proteins [68]. Ninfali et al. [69] analysed molecular mechanisms of antiviral effects exerted by apigenin, vitexin, quercetin, rutin, and naringenin. They mainly inhibited viral neuraminidase, proteases, and DNA/RNA polymerases, as well as modified different viral proteins [69]. Moreover, flavonoids can also induce the host immune response, regulate the inflammatory response, and block the receptor–virus combination, thereby reducing virus load [70]. Tang et al. [71] explored the antiviral activities of flavonoids present in the plant *Scutellaria barbata* used in traditional Chinese medicine. They assessed the inhibitory activities of *S. barbata* extracts against HIV-1 protease (HIV-1 PR) and SARS-CoV-2 viral cathepsin L protease (Cat L PR). They determined the inhibitory activities of nine flavonoids (luteolin, hispidulin, scutellarein, apigenin, naringenin, eriodictyol, scutellarin, baicalin, and wogonin) and four extracts of *S. barbata* (SBW, SB30, and SB60) against HIV-1 PR and Cat L protease. All extracts as well as all tested flavonoids inhibited HIV-1 PR, with IC_50_ values in the range of 0.006 to 0.83 mg/mL. Six flavonoids (scutellarein, apigenin, hispidulin, naringenin, eriodictyol, and scutellarin) demonstrated 10–37.6% inhibition of Cat L PR at a concentration of 0.1 mg/mL. However, the poor buffer solubility of the samples may have lowered the bioavailability for Cat L PR [71].

Flavonoids (Figure 3) can inhibit fungal growth through various mechanisms, such as plasma membrane disruption, the induction of mitochondrial dysfunction, and inhibiting cell wall formation, cell division, RNA and protein synthesis, and the efflux-mediated pumping system [72]. Salazar-Aranda et al. [73] evaluated the antifungal and antioxidant activity of various polyphenolic compounds. Myricetin and baicalein inhibited the growth of all species tested, but the strongest effect was exhibited against *Candida glabrata*, with a minimum inhibitory concentration (MIC) value lower than that of fluconazole. The MIC values against *C. glabrata* for myricitrin, luteolin, quercetin, 3-hydroxyflavone, and fisetin were similar to those of fluconazole. Polyphenolic compounds with antioxidant activity revealed the most significant activity against *C. glabrata*, whereas *Candida albicans* was less susceptible to these flavonoids.

Penna-Coutinho et al. [74] investigated both commercial drugs containing flavonoids—Accuvit^®^, Ginkgo^®^, and Soyfit^®^—and the standard flavonoids hesperidin, quercetin, and genistein against blood cultures of chloroquine-resistant *Plasmodium falciparum*, as well as chloroquine, a reference antimalarial. They measured the reduction in parasite growth in mice with *Plasmodium berghei* malaria and in vitro against chloroquine-resistant *P. falciparum.* Accuvit^®^ turned out to be the most active drug in vitro (IC_50_ 5 μg/mL), whereas Soyfit^®^ revealed only partial activity (IC_50_ 13.6 μg/mL), and Ginkgo^®^ (IC_50_ 38.4 μg/mL) was inactive. As far as in vitro studies are concerned, Accuvit^®^ and quercetin caused the highest reduction in *P. berghei* parasitaemia (63% and 53%, respectively) on day 5 after parasite inoculation. It was concluded that not only flavonoids contributed to the antimalarial activity of Accuvit^®^ but also synergistically acting multivitamins.

## 3. Synthesis

Fluorinated flavonoids and chalcones are prepared via two routes: from fluorinated precursor substrates and by structure modification using fluorination reaction.

### 3.1. Synthesis from Precursors

**Figure 4:**
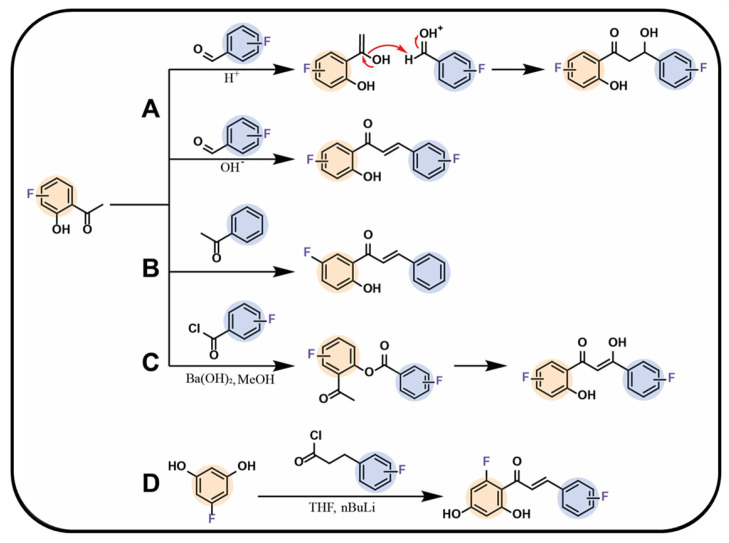
Selected condensation reaction pathways for chalcones: (**A**) chalcone condensation by Claisen-Schmidt reaction in different pH, (**B**) chalcone condensation of two aromatic ketones, (**C**) chalcone condensation using aromatic ketone and acid chloride, (**D**) chalcone synthesis proposed by Tsunekawa et al. [91]; THF—tetrahydrofurane, BuLi—butyllithium.

One of the most commonly used methods for obtaining fluorinated chalcone and flavonoid derivatives is their synthesis from fluorinated precursors. Chalcones can be relatively easily synthesised through the Claisen–Schmidt reaction (aldol condensation). In this process, an aromatic aldehyde reacts with an aromatic ketone in a basic medium, leading to the formation of an *α*,*β*-unsaturated carbonyl compound—chalcone. When the reaction is conducted in an acidic medium, the resulting compound has a saturated *α*,*β*-bond. In this approach, the starting substrates, aldehydes or ketones, should contain the fluorine functionalities at certain positions of the chemical structure. The reaction mechanism is shown in Figure 4A [75]. A similar approach was proposed by Conti et al. [76], who obtained fluorinated flavones through the reaction of aromatic ketone derivatives in the presence of barium hydroxide in methanol. The yield of this synthesis method ranged between 70% and 95% (Figure 4B) [76]. Variations of this reaction are also known and rely on the use of acid chlorides instead of aldehydes [75,76,77,78,79,80,81,82,83,84,85,86,87,88]. Another commonly encountered mechanism relies on the reaction between ortho-hydroxyacetophenones and benzoyl chlorides. This reaction follows a different pathway, initially leading to the formation of an ester intermediate, which, subsequently under basic conditions, undergoes rearrangement into chalcone (Figure 4C) [78,89,90]. The following method that leverages acid chlorides for the synthesis of fluorinated chalcones was presented by Tsunekawa et al. [91]. In this approach, a fluorinated benzene derivative—3-fluoro-1,5-dimethoxybenzene—was used along with an acid chloride containing the future B-ring and a two-carbon alkyl chain in the presence of butyllithium in tetrahydrofuran (Figure 4D) [91].

**Figure 5:**
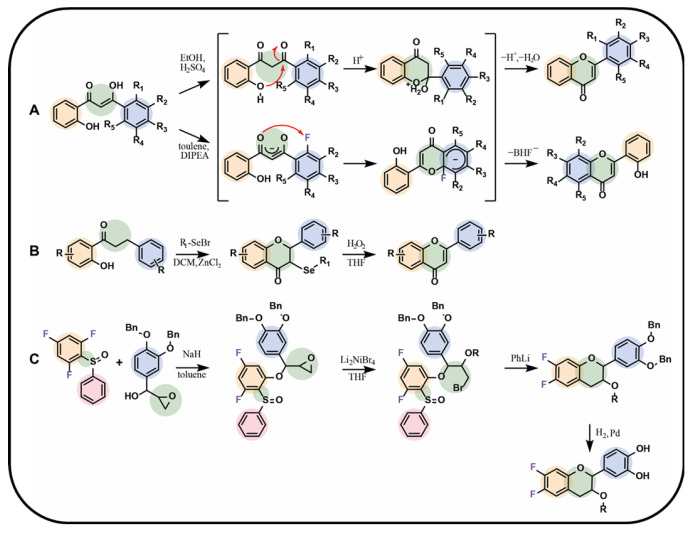
Selected syntheses of fluorinated flavonoids: (**A**) chalcone cyclisation in different media by Shcherbakov et al. [89], (**B**) chalcone cyclisation with selenium bromide derivative as a catalyst by Zhu et al. [78], (**C**) flavane synthesis by Stadlbauer et al. [93]; Bn—benzyl group, DCM—dichloromethane, DIPEA—*N*,*N*-diisopropylethylamine, THF—tetrahydrofuran, R—fluorine, hydrogen atom, or alkyl group, Li_2_NiBr_4_—dilithium tetrabromonickel, PhLi—phenyllithium.

The desired chalcone can be subjected to a cyclisation reaction with C-ring formation in the final flavonoid product structure. The reactions conducted by Shcherbakov et al. provided insights into this mechanism, particularly regarding the behaviour of fluorine when substituted at the C2′ position. Depending on the reaction conditions, the B-ring of the chalcone could be either transformed into the A-ring or remain as the B-ring of the final flavonoid (Figure 5A) [89]. Another method of the chalcone ring closure involves the use of iron(III) chloride, an excess of N-fluorobenzenesulfonimide (NFSI) [90], selenium-based catalyst such as selenium bromide (Figure 5B) [78], and molecular iodine in dimethyl sulfoxide at 140 °C [92] or pyridine at 90 °C [91]. Conti et al. [76] proposed C-ring flavonoid formation using hydrogen peroxide and a 5% sodium hydroxide solution in ethanol. For all the aforementioned reactions, the presence of a hydroxyl group at the C2 position of the A-ring in a precursor chalcone substrate is necessary.

Stadlbauer et al. [93] presented a unique method for the synthesis of flavonoids, avoiding the chalcone intermediates (Figure 5C). In this approach, a fluorinated precursor obtained by the coupling of 1,3,5-trifluoro-2-sulfinylbenzene with the epoxide-containing compound was applied in the presence of sodium hydride in toluene. The intermediate epoxide was then reduced with dilithium tetrabromonickelate(II) solution in tetrahydrofuran, and the C-ring was formed in the presence of phenyllithium in tetrahydrofuran. The limiting step was a coupling reaction with a yield of only 34%, whereas the remaining steps led to the products with yields between 90% and 95%. The procedure led to the fluorinated flavane as a final product [93].

**Figure 6:**
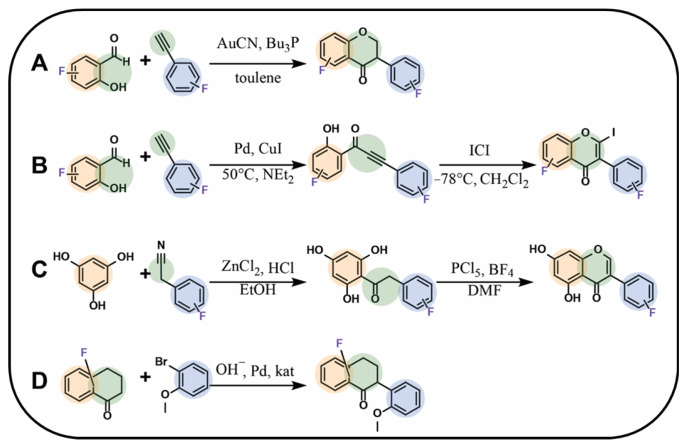
Selected syntheses of fluorinated isoflavonoids: (**A**) isoflavanone synthesis using aromatic aldehyde and phenylacetylene by Amato et al. [94], (**B**) isoflavone synthesis using aromatic aldehyde and phenylacetylene by Zhu et al. [78], (**C**) isoflavone synthesis by Yang et al. [95], (**D**) 1-carbaisoflavanones synthesis by Caleffi et al. [96]; DMF—dimethylformamide, Bu_3_P—tributylphosphine, NEt_3_—trimethylamine, ICl—iodine monochloride.

Isoflavonoids can be obtained following various synthetic approaches. An interesting method was proposed by Amato et al. [94], who obtained fluorinated isoflavanones using a fluorinated derivative of hydroxyaldehyde and a corresponding fluorinated derivative of phenylacetylene under microwave irradiation using gold(I) catalysts such as gold(I) cyanide and tributylphosphine in toluene, with the yield ranging between 20% and 40% (Figure 6A) [94]. Meanwhile, Zhu et al. [78], using the same type of substrates but different catalysts, obtained fluorinated isoflavone using iodine monochloride in the cyclisation reaction (Figure 6B). A synthesis similar to the two abovementioned was proposed by Yang et al. [95], leading this time to isoflavone as the product. The process involved coupling 1,3,5-trihydroxybenzene with a fluorocyanobenzene derivative in the presence of zinc(II) chloride and dry hydrogen chloride gas in anhydrous diethyl ether, leading to the formation of a fluorinated chalcone with yields ranging between 40% and 60%. In the next step, the cyclisation process was carried out in dimethylformamide with boron trifluoride at 0 °C. Then, phosphorus pentachloride was added, resulting in the formation of the desired product (Figure 6C) [95].

Caleffi et al. [96] proposed a mechanism for the synthesis of fluorinated 1-carbaisoflavanones with the use of a fluorinated precursor bearing future product A- and C-rings with a substituted bromobenzene. The B-ring in the final product is localised at the C3 position. The reaction was carried out in dioxane/water (4:1) with a palladium catalyst in a basic environment and tri-*tert*-butylphosphonium tetrafluoroborate under microwave irradiation for 1 h, and the yield ranged from 85% to 92% (Figure 6D) [96].

### 3.2. Structure Modification by Fluorination

**Figure 7:**
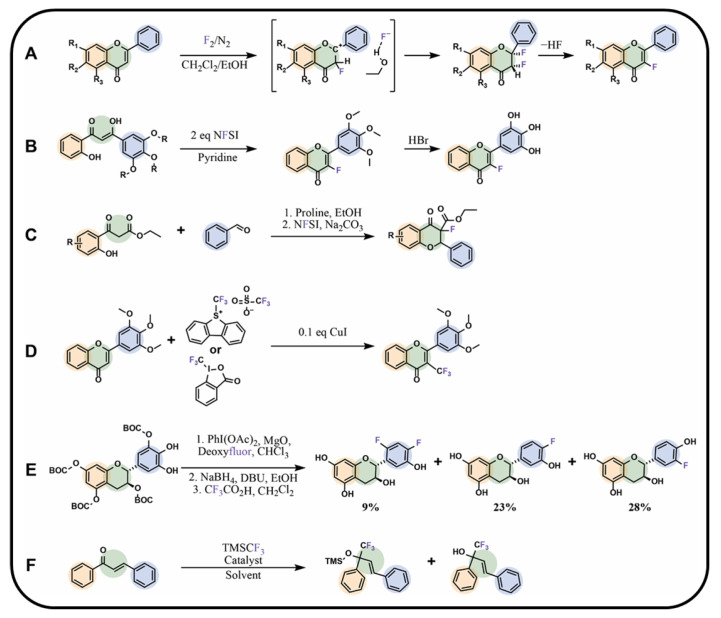
Modifications of flavonoids or chalcones with groups containing fluorine: (**A**) flavonoid fluorination with molecular fluorine, (**B**) chalcone fluorination with NFSI accompanied by simultaneous cyclisation to flavonoid, (**C**) fluorinated flavonoid synthesis by Cui et al. [99], (**D**) flavonoid trifluoromethylation using Togni or Umemoto reagents, (**E**) flavonoid deoxyfluorination [100], (**F**) chalcone trifluoromethylation proposed by Dong et al. [102]; BOC—*tert*-butyloxycarbonyl group, DBU—1,8-diazabicyclo[5.4.0]undec-7-ene, NFSI—*N*-fluorobenzenesulfonimide, TMS—trifluoromethyltrimethylsilane, R—hydrogen atoms, alkyl or hydroxyl groups, CF_3_CO_2_H—trifluoroacetic acid.

The fluorine atom can be introduced into the flavonoid molecule using molecular fluorine gas. However, this reaction presents several challenges. First, fluorine is highly toxic, and second, the reaction is strongly exothermic, making the process difficult to control. This issue can be addressed by diluting fluorine in an inert gas (N_2_) and conducting the reaction at low temperatures. The weak bond between the two fluorine atoms can be stabilised using a relevant polar solvent. However, gaseous fluorine can be displaced from the solution when the solvent is too polar. According to the studies by Vints and Rozen, a chloroform–ethanol mixture provides the optimal conditions for fluorination [97]. Alcohol acts as a good acceptor for the “negative pole” of the halogen molecule, while chloroform enhances the solubility of fluorine gas in the solution. Unlike other halogens, fluorine cannot form a bridged fluoronium ion with an olefin through a C2C3 double bond. Instead, an intermediate species containing fluorine and an unstable *α*-fluorocarbenium ion is formed. The anion connects at the C2 position in flavones, whereas the fluorine connects at the C3 position. The unstable ion subsequently converts into a difluorinated flavan. By eluting the flavan on silica gel with a petroleum ether/ethyl acetate mixture or placing it in a solution of boron trifluoride diethyl etherate, the elimination of a hydrogen fluoride molecule can be induced, yielding the fluorinated flavone. The yields of individual reaction steps varied significantly depending on the position of the other substituents (R_1_, R_2_, and R₃) and ranged from 49% to 95% (Figure 7A) [97].

Alshammeri et al. [90] did not use a fluorinated chalcone as a precursor in their synthesis. Instead, they utilised a *β*-diketone chalcone derivative with a hydroxyl group at the C2 position in the fluorination reaction using NFSI in a two-fold excess relative to the substrate. As a result, fluorine substitution at the C3 position of the molecule was accompanied by a simultaneous cyclisation reaction into a flavone. When NFSI was used in a 1:1 ratio relative to the chalcone, only fluorine substitution occurred, with no cyclisation observed. In such cases, C-ring closure was carried out using iron(III) chloride. The resulting fluoroflavonoid, 3-fluoro-3′,4′,5′-trimethoxyflavone, was demethylated using hydrobromic acid, yielding the final product, 3-fluoro-3′,4′,5′-trihydroxyflavone. Pyridine as a solvent was found to be superior to dichloromethane/acetonitrile and led to the product with the highest yield (Figure 7B) [90]. Fluorine can also be introduced at the C3 position using the Oxa-Michael electrophilic addition with NFSI, ensuring a higher yield. However, the resulting product does not retain the C2C3 double bond [98]. Cui et al. also applied NFSI in their study (Figure 7C) [99].

A reagent known as deoxyfluor—bis(2-methoxyethyl)aminosulfur trifluoride—was studied for monofluorination of flavonoids with a catechol group, and it was demonstrated that the fluorinated ring is always the B-ring. During the reaction, the hydroxyl group was substituted by fluorine. The main challenge of this reaction is the high reactivity of deoxyfluor, which results in a mixture of mono- and difluorinated derivatives with fluorine in various positions. In addition to hydroxyl substitution, hydrogen substitution may also occur. As a result, the post-reaction mixture must be separated by chromatography (Figure 7E) [100].

The trifluoromethyl group can be introduced directly into the flavonoid structure using CF_3_SiMe_3_ dissolved in tetramethylammonium fluoride. In this process, in case of isoflavones, the group attaches exclusively at the C2 position of the C-ring, replacing the double bond. In contrast, in the case of flavones, the group attaches to the C4 carbon, simultaneously reducing the carbonyl group to a hydroxyl group, while the double bond remains intact. The product of this reaction is flavol. The use of protecting groups for hydroxyl groups is necessary [101]. Another method for introducing the trifluoromethyl group into the flavonoid structure involves using other halogen derivatives (typically iodine) and performing a substitution reaction. Iodine can be displaced from the molecule by FSO_2_CF_2_CO_2_Me in the presence of a copper(I) iodide catalyst [101]. An interesting method for introducing the trifluoromethyl group into the chalcone structure was proposed by Dong et al. [102], who incorporated it directly into the structure using caesium carbonate as a catalyst and trifluoromethyltrimethylsilane (TMSCF_3_) as the fluorinating agent, targeting the carbon of the carbonyl group. The use of caesium carbonate, potassium carbonate, potassium hydroxide and potassium *tert*-butoxide led to desired products with 94%, 72%, 83%, and 71% yields, respectively. The choice of solvent can also influence the structure of the obtained product. Dichloromethane promoted the synthesis of the tetramethylsilane (TMS)-containing compound, whereas dimethylformamide and dimethylacetamide (DMA) led to the formation of a desililated product (Figure 7F) [102]. Alshammeri [103] also attempted the trifluoromethylation of a *β*-diketone chalcone using Togni I, Togni II, or Umemoto fluorinating agents. Unfortunately, modifying the chalcone with any of these reagents was unsuccessful, regardless of the solvent used. However, flavonoids with a closed C-ring could be successfully modified using Togni I or Umemoto reagents in the presence of copper(I) iodide in dimethylformamide. The resulting product, 3-trifluoromethyl-3′,4′,5′-trimethoxyflavone, was obtained with an average yield of 20% (Figure 7D) [103].

## 4. Fluorinated Derivatives of Flavonoids and Chalcones of Potential Applications in Medicine

Fluorinated flavonoids constitute a significant area of research focused on discovering compounds that exhibit diverse properties. Inextricably bound to this topic are chalcones, which serve as key precursors in flavonoid synthesis.

### 4.1. Antibacterial, Antifungal and Antiparasitic Properties

As previously mentioned, flavonoids alone exhibit antibacterial activity, with several mechanisms proposed. These include the inhibition of bacterial cell membrane synthesis and biofilm formation, the disruption of the electron transport chain and ATP production, and the inhibition of specific enzymes. Chalcones, on the other hand, display strong antibacterial activity, especially against multidrug-resistant bacteria, by targeting enzymes such as DNA gyrase B, MurA transferase, and efflux pumps. Finally, both flavonoids and chalcones influence the host organism by modulating immune and inflammatory responses [64,104,105].

**Figure 8:**
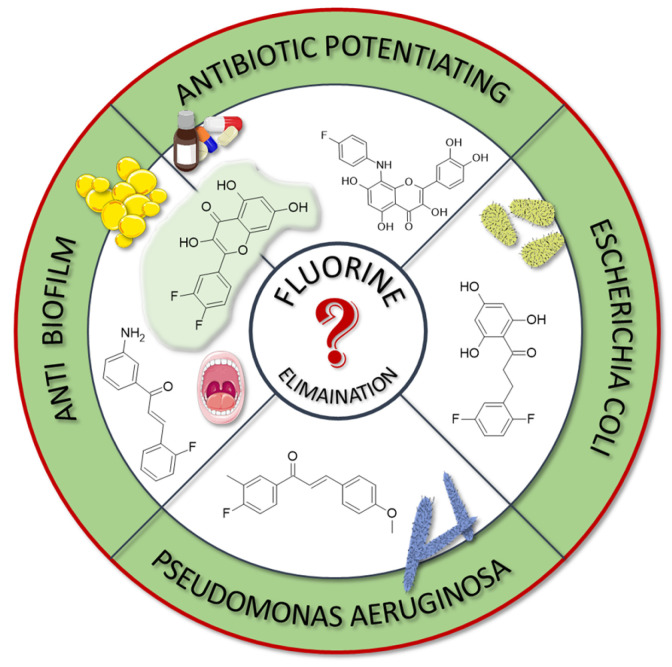
Fluorinated derivatives of flavonoids and chalcones of potential antimicrobial properties.

**Figure 9:**
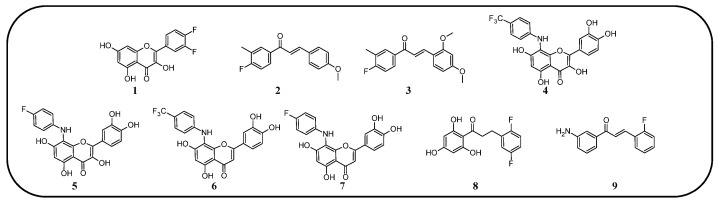
Flavonoids, chalcones, and their fluorinated derivatives (**1**–**9**) with antibacterial properties.

In many studies presented below, fluorinated derivatives of flavonoids and chalcones revealed potential antimicrobial properties (Figure 8, Table 1). The continuous evolution of treatment-resistant bacterial strains directs scientists to search for compounds that microorganisms have not yet adapted to and rendered harmless. In this regard, fluorinated flavonoids reveal some prospective antimicrobial potential. The influence of B-ring fluorination in this context was evaluated by Kho et al. [106] in a series of tests on a fluorinated derivative of quercetin, 3′,4′-difluoroquercetin **1** (Figure 9). The tests were conducted on various Gram-positive and Gram-negative bacterial strains. Compound **1** exhibited interesting antibacterial properties against Gram-positive bacteria, such as *Staphylococcus aureus* and *Enterococcus* sp. (MIC values ranging from 8 to 32 mg/L), compared to Gram-negative bacteria (MIC > 128 mg/L). Further research on *S. aureus* biofilms revealed good antibiofilm activity, with IC_50_ values ranging from 1.8 to 5.3 mg/L. The best activity was observed against methicillin-resistant *S. aureus* (MRSA), while compound **1** was least effective against vancomycin-intermediate *S. aureus*. The checkerboard synergy test evaluated the antibacterial activity of **1** in combination with the following antibiotics: aminopenicillin, ceftazidime, cefepime, meropenem, and vancomycin. The best antimicrobial properties (MIC_50_ 2 mg/L) were observed against *P. aeruginosa* for the combination of ceftazidime and **1**. The efflux activity of carbapenem-resistant *P. aeruginosa* revealed that **1** could act as an inhibitor of transmembrane efflux pumps, but it was slightly weaker than the reference, carbonyl cyanide 3-chlorophenylhydrazone (CCCP). Next, in vivo tests were conducted on mice infected with methicillin-resistant *P. aeruginosa* or carbapenem-susceptible *P. aeruginosa*. The results showed significantly higher survival rates in animals treated with a combination of ceftazidime and **1** (10 and 40 mg/kg body mass) compared to those treated with the antibiotic alone or left untreated [106]. Other bacterial efflux pump inhibitors were synthesised by Hurtová et al. [107]. They studied quercetin and luteolin modified with fluorinated aniline (**4**–**7**). The aniline group was substituted at position C8, with a trifluoromethyl group or a fluorine atom located in the para or meta positions relative to the nitrogen atom. Assays conducted on MRSA with gentamicin (1 mg/L) showed that the best antimicrobial properties were exhibited by 8-(4-(trifluoromethyl)anilino)quercetin (**4**), 8-(4-fluoroanilino)luteolin (**7**), and 8-(4-(trifluoromethyl)anilino)luteolin (**6**), with MIC values of approximately 8 μM. In comparison, the combination of gentamicin at breakpoint concentration with unmodified quercetin showed an MIC > 200 μM, while the combination with luteolin revealed an MIC equal to 84,7 μM. The same compounds also exhibited the most significant enhancement of the antibacterial properties of erythromycin, with MIC values ranging from 15 to 25 μM. Monofluorinated compounds were also tested for their ability to inhibit bacterial efflux pumps. For example, 8-(4-fluoroanilino)quercetin at 50 μM (**5**) exhibited the same activity as CCCP at 100 μM, while compound **7** displayed even better properties. Notably, the monofluoroflavonoid derivatives were many times more effective at lower concentrations than CCCP. Further studies on modulating erythromycin resistance in MRSA revealed that 8-(4-(trifluoromethyl)anilino)quercetin (**4**) and –luteolin (**6**) can inhibit ribosomal methyltransferase. However, the precise mechanism of action of the studied compounds remains unclear [107]. Trifluoromethyl substituents were reported to be more beneficial for increasing flavonoid antibacterial activity than chlorine. Shoaib et al. [108] synthesised a flavone derivative with a trifluoromethyl substituent at the C4′ position and tested it against *Bacillus subtilis*, *S. aureus*, and *P. aeruginosa*. The MIC values for this compound were 12.5, 25, and 25 µg/mL, respectively, whereas the unmodified flavonoid was studied at the concentrations of 25, 37.5, and 25 µg/mL. The addition of a methoxy group to a fluorinated flavone at the C7 position significantly weakened the antibacterial properties. In contrast, a derivative with a trifluoromethyl group at C3′ and a bromine atom at C6′ exhibited MIC values of 12.5, 6.25, and 6.25 µg/mL, respectively. These values were identical to those obtained for ciprofloxacin, except for *B. subtilis*, where the antibiotic was twice as effective. Additionally, chlorinated flavonoid derivatives were also tested but displayed significantly weaker antibacterial activity. These results suggest that trifluoromethyl-substituted derivatives exhibit superior antibacterial properties compared to their chlorine-containing counterparts [108].

Selected plants, such as *Glycyrrhiza inflata* and *Piper sanctum*, naturally produce chalcones with antibacterial properties, e.g., licochalcone A. For this reason, chalcone derivatives are frequently studied for their antibacterial properties. Based on the study by Vane et al. on the antibacterial properties of C4′-fluorinated chalcones, it can be concluded that the addition of fluorine and methoxy groups could be crucial for their antibacterial activity. The study was conducted on *S. aureus* (MCC2408), *B. subtilis* (MCC2010), *E. coli* (MCC2412), and *P. aeruginosa* (MCC2080). Notably, the compound containing fluorine along with two methoxy groups (at the C2′ and C4′ positions) (**3**) exhibited superior antibacterial properties compared to streptomycin. Meanwhile, the compound with only one methoxy group at the C4′ (**2**) position demonstrated the highest activity against the *P. aeruginosa* strain. Compounds with a higher number of fluorine atoms or methoxy groups in the B-ring at other positions displayed significantly weaker or comparable antibacterial activity to streptomycin [77].

A series of studies on 2,4,6-trimethoxy or hydroxyl and non-/mono- or difluoro-substituted chalcone derivatives was conducted by Burmaoglu et al. [109]. Among all the tested compounds, only the compound with three hydroxyl groups in the A-ring (C2, C4, and C6) and two fluorine atoms in the B-ring (C2′ and C5′) (**8**) exhibited antibacterial properties (MIC 7.8 µg/mL) against *S. aureus*. Interestingly, these compounds, including **8**, did not reveal promising activity against *S. pyogenes*, *E. faecalis*, *E. coli*, or *P. aeruginosa* (MIC values ranged between 30 and 125 µg/mL). In addition, ampicillin, as an antibiotic, presented moderate activity against *E. faecalis*, *P. aeruginosa*, *and E. coli*, with MIC values of 62.5 µg/mL, 31.25 µg/mL, and 3.9 µg/mL, respectively [109].

A very interesting study was conducted by Lima et al. [110]. They evaluated 3-amino-2′-fluorochalcone (**9**) suspended in a mucoadhesive hydrogel (at a concentration of 300 µg/mL) for its antimicrobial activity against *Aggregatibacter actinomycetemcomitans*, *Fusobacterium periodonticum*, *Prevotella intermedia*, *Porphyromonas gingivalis*, and *Tannerella forsythia*—pathogens associated with periodontal disease and peri-implant infections. In vitro tests against these bacteria revealed MIC values ranging from 7.8 to 31.25 μg/mL for bare compound **9**, and from 3.9 to 15.6 μg/mL for its hydrogel formulation, with SI between 8 and 32. For comparison, standard antibiotics, such as metronidazole and amphotericin B, exhibited significantly stronger antibacterial activity, with MIC values between 0.195 and 0.5 μg/mL. Further study, which was conducted on titanium conical implantable screws, demonstrated the excellent antibiofilm properties of the hydrogel formulation containing **9**, which was comparable to 0.12% chlorhexidine. Toxicity assays performed on *Galleria mellonella* larvae infected with *P. gingivalis* indicated the low toxicity of **9**, maintaining high larval survival rates [110].

**Table 1:** Short summary of the antibacterial properties of fluorinated derivatives of flavonoids and chalcones.

To Sum Up
Pros
Fluorinated flavonoids and chalcones exhibit good antibacterial activity, with MIC values comparable to traditional antibiotics [109]. Fluorinated flavonoids enhance the antibacterial potential of antibiotics by interacting with efflux pumps and demonstrate a synergistic effect, even against antibiotic-resistant bacteria [106,107]. 3′,4′-Difluoroquercetin exhibits good antibiofilm properties against *S. aureus* [106].
**Cons**
Certain mechanisms allow bacteria to eliminate fluorine from the molecule, thereby weakening the effect of the xenobiotic [111].

**Figure 10:**
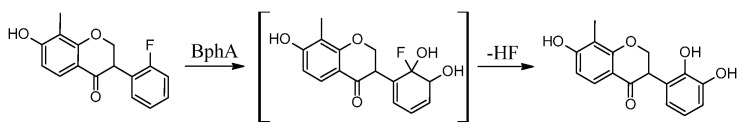
Proposed mechanism of flavonoid defluorination in microorganisms.

Interestingly, certain natural mechanisms were found in microorganisms that are related to the removal of a fluorine atom from the structure of a fluorinated flavonoid. The study by Seeger et al. [111] was conducted on recombinant *E. coli* cells expressing the biphenyl-2,3-dioxygenase (BphA) genes of *Burkholderia* sp. (strain LB400). It was found that 2′-fluoro-7-hydroxy-8-methylisoflavanone was enzymatically converted to 7,2′,3′-trihydroxy-8-methylisoflavone (Figure 10) [111].

Flavonoids and chalcones exhibit antifungal activity by disrupting cell membranes and mitochondria, interfering with cell division, and inhibiting protein and RNA synthesis [72,112]. Similarly, these compounds also demonstrate significant antiparasitic properties. Their mechanisms of action are varied and include the induction of oxidative stress in parasitic cells, inhibition of crucial parasitic enzymes (e.g., proteases, reductases), disruption of cellular membranes and mitochondrial integrity, and modulation of the host immune response. Moreover, flavonoids have been reported to induce parasite paralysis, disturb calcium homeostasis, alter glucose and glycogen metabolism, and increase nitric oxide synthase activity [74,96,112].

**Figure 11:**
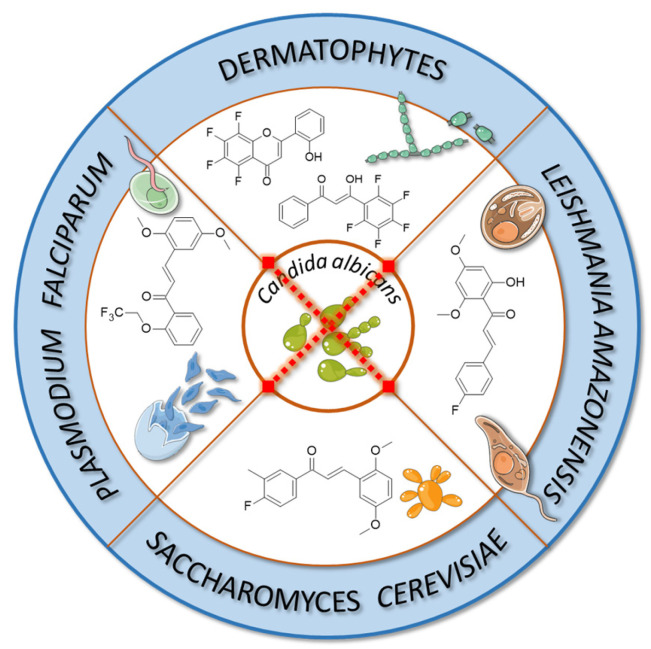
Fluorinated derivatives of flavonoids and chalcones of potential antifungal and antiparasitic properties.

**Figure 12:**
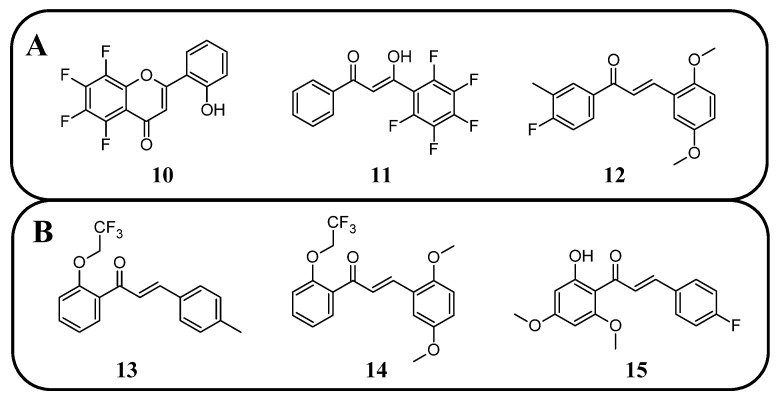
Fluorinated flavonoids and chalcones with (**A**) antifungal properties (**10**–**12**) and (**B**) antiparasitic activity (**13**–**15**).

Fluorinated derivatives of flavonoids and chalcones revealed potential antifungal and antiparasitic properties (Figure 11, Table 2). There are not many studies on the antifungal properties of fluorinated flavonoids and chalcones. One such study, presented by Shcherbakov et al. [89], clearly demonstrated that flavones with a fluorinated B-ring exhibited little to no antifungal activity. In contrast, fluorination of the A-ring **10** (Figure 12) resulted in compounds with strong antifungal properties. However, these compounds were not universally effective, as they specifically targeted species such as *Trichophyton tonsurans*, *T. rubrum*, and *Epidermophyton floccosum*. These are the fungi primarily responsible for skin fungal infections—dermatophytes. Fluorinated chalcones also exhibited excellent antifungal properties, particularly the chalcone containing five fluorine atoms in the B-ring (**11**), with an MIC < 1.56 µM for nearly all tested species. However, no clear correlation was observed between the number of fluorine atoms and their fungistatic effect. Additionally, the compounds did not reveal activity against *Candida albicans*. This study underscores the promising antifungal potential of fluorinated chalcones and the selective activity of flavonoids with fluorinated A-rings, suggesting targeted applications rather than broad-spectrum use [89].

Antifungal tests conducted using chalcone derivatives fluorinated at the C4′ position showed that none of them exhibited better activity against *C. albicans* (MCC1439) than fluconazole. However, the chalcone derivative with methoxy groups at the C3′ and C6′ positions, a methyl group at C3, and a fluorine atom at C4 (**12**) demonstrated robust activity against *S. cerevisiae* (MCC1033), being twice as effective as fluconazole [77]. Unfortunately, the studies do not contain information about the selectivity of these compounds in the presence of healthy cells.

The previously mentioned study by Lima et al. indicates that the synthesised compound **9** (Figure 10), suspended in a hydrogel (300 µg/mL), exhibits good antibiofilm properties against *C. albicans* (MIC 7.8 µM), comparable to 0.12% chlorhexidine. This property was investigated on titanium conical implantable screws [110].

Fluorinated chalcones are also being researched for their antiparasitic properties. Devi et al. [85] studied 2,2,2-trifluoroethoxychalcone and 2-fluoroethoxychalcone derivatives against *P. falciparum* (3D7). The key finding was that monofluoroethoxy derivatives consistently exhibited up to five-fold greater parasite-killing ability compared to their corresponding trifluoroethoxy derivatives. However, two trifluoroethoxy derivatives stood out for their superior activity: (*E*)-3-(4-methylphenyl)-1-(2-(2,2,2-trifluoroethoxy)phenyl)prop-2-en-1-one **13** (Figure 12) (IC_50_ 9.4 µM) and (*E*)-3-(2,5-dimethoxyphenyl)-1-(2-(2,2,2-trifluoroethoxy)phenyl)prop-2-en-1-one (**14**) (IC_50_ 2.2 6 µM). In contrast, other trifluoro derivatives failed to achieve IC_50_ values below 10 µM. The selectivity assay conducted on Vero cells revealed that these two compounds had the highest selectivity indexes (8.6 and 8.2, respectively). No strict correlation was observed between the presence of the fluoroethoxy group in the chalcone structure and its antiparasitic properties [85].

Boeck et al. [86] evaluated the properties of fluorinated chalcones for combating *Leishmania amazonensis*, a flagellate parasite transmitted by mosquitoes that causes leishmaniasis (Dum-Dum fever). The compounds were tested in vitro in cultures containing insect-stage promastigotes and intramacrophage amastigotes of *L. amazonensis* (Josefa strain). Among the tested compounds, 4′-fluoro-2-hydroxy-4,6-dimethoxychalcone (**15**) was the only fluorinated chalcone examined. Its IC_50_ values were ~0.8 µM against promastigote forms and 4.3 µM against amastigote forms. Additionally, the compound showed minimal toxicity to macrophages (IC_50_ > 100 µM), making it one of the most promising antiparasitic agents among those studied [86].

### 4.2. Antiviral Properties

Natural chalcones and their derivatives exhibit potent antiviral properties by selectively inhibiting viral enzymes such as lactate dehydrogenase, fumarate reductase, protein kinases, and integrase/protease. In contrast, the antiviral activities of flavonoids are not as extensively documented. These compounds show limited ability to interact with viral neuraminidase, proteases, and DNA/RNA polymerases. Nevertheless, both flavonoids and chalcones appear to be promising agents in combating HIV-1 and SARS-CoV-2 infections. In addition to their direct antiviral effects, these compounds may also enhance the host immune response [71,105].

**Figure 13:**
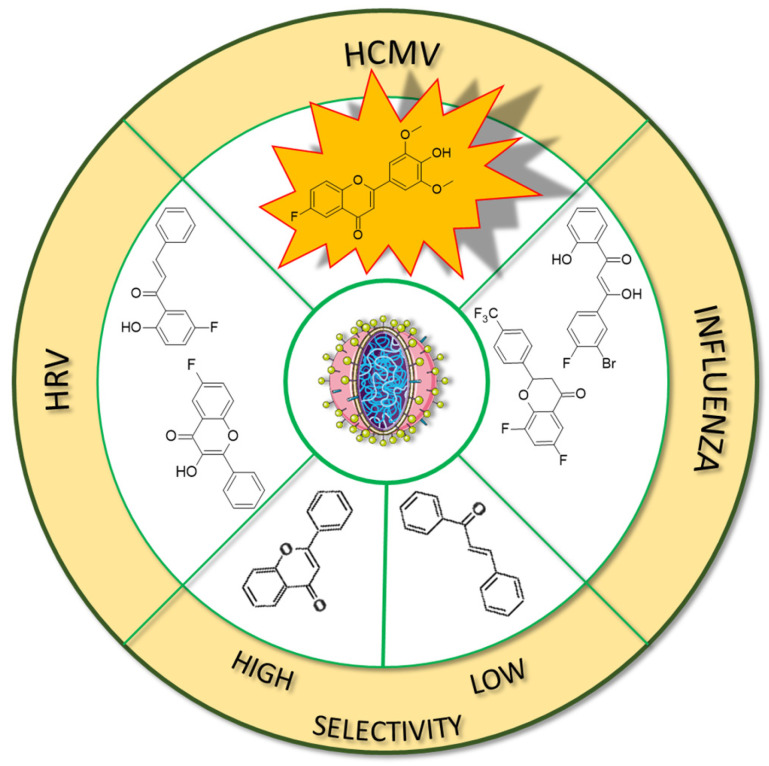
Fluorinated derivatives of flavonoids and chalcones of potential antiviral properties. HRV—human rhinovirus; HCMV—human cytomegalovirus.

**Figure 14:**
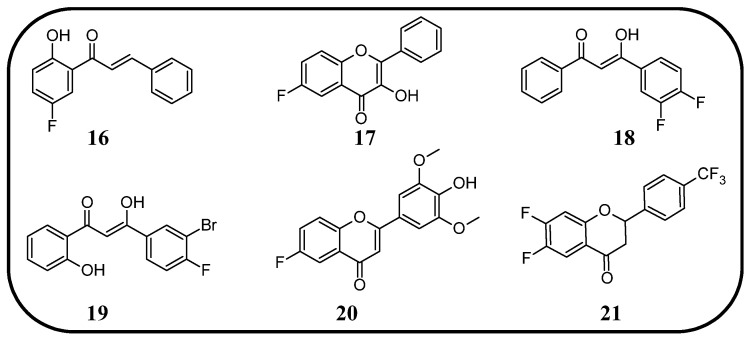
Flavonoids, chalcones, and their fluorinated derivatives (**16**–**21**) with antiviral properties.

In many studies presented below, fluorinated derivatives of flavonoids and chalcones revealed potential antiviral properties (Figure 13). The introduction of fluorine into chalcone scaffolds is an interesting strategy for improving antiviral activity. Many studies have explored the antiviral potential of fluorinated chalcones against respiratory viruses. The series of compounds tested by Conti et al. [76] included fluoro-substituted flavonoids, 2-styrylchromones, and chalcones. The antiviral properties were assessed on HeLa cell lines infected with human rhinovirus (HRV) serotypes 1B and 14. It turns out that fluorinated compounds (including flavonoids with a fluorine atom at position C6) exhibit weak activity against HRV 1B. The most promising compound was the chalcone 1-(5-fluoro-2-hydroxyphenyl)-3-phenylpropen-1-one **16** (Figure 14, Table 3), with a concentration required to reduce the HRV 1B virus plaque count by 50% at 5.84 µM. The 6-fluoro-3-hydroxyflavone (**17**) demonstrated the best activity against HRV 14 serotype A (IC_50_ 5.56 µM). This compound also showed high cytotoxicity, with CC_50_ values of 25 µM. In addition, the tested flavonoids and chalcones caused a reduction in the size of viral plaques (from 50% to 70%) for both HRV serotypes, suggesting a slowdown in viral replication kinetics. In the case of fluorostyrylchromones, no significant size changes were observed, or they were negligible, whereas the compound BW683C (a dichloroflavan with strong antiviral properties) had an IC_50_ of approx. 0.025 µM against HRV 1B and presented no activity against HRV 14 [76,113]. Other fluorinated chalcones, particularly 1,3-diaryl-1,3-diketones, were tested by Shcherbakov et al. [89]. The analysis focused on their interaction against influenza type A/Puerto Rico/8/34 (H1N1) in infected Madin–Darby canine kidney (MDCK) cell lines. The B-ring was fluorinated in the tested compounds. Low selectivity values were observed for fluorinated compounds, especially for fluorinated flavones (selectivity index below 5). A similar trend was observed for chalcones containing four fluorine atoms in the B-ring and chalcones with methylated hydroxyl groups. Interestingly, the IC_50_, indicating the reduction in viral production, was lowest among chalcones with the fewest fluorine atoms. The compound containing fluorine atoms at C3′ and C4′ positions had an IC_50_ value of 7 µM and SI of 16 (**18**), while the compound with fluorine at C4′ and bromine at C3′ presented an IC_50_ of 6 µM with an SI of 33 (**19**). The IC_50_ values of other chalcones ranged between 10 and 16 µM, still significantly outclassing fluorinated flavonoids, which had IC_50_ values between 200 and 300 µM. Regarding cytotoxicity towards MDCK cells, a fully fluorinated B-ring posed the most significant risk, with a CC_50_ of 57 µM, and the chalcone with the C3, C4, and C5 positions fluorinated had a CC_50_ of 43 µM. The CC_50_ values for fluorinated chalcones ranged between 43 and 300 µM, while those for flavonoids were around 1000 µM. For comparison, ribavirin, an antiviral drug, showed a CC_50_ of approx. 2000 µM, an IC_50_ of 36 µM, and a selectivity index > 59 [89].

Fluorinated flavonoids were tested against influenza viruses and human cytomegalovirus (HCMV). Tricin (4′,5,7-trihydroxy-3′,5′-dimethoxyflavone) and its fluorinated derivative 6-fluoro-4′-hydroxy-3′,5′-dimetoxyflavone (**20**) were compared to ganciclovir by evaluating antiviral activity on human embryonic lung (HEL) fibroblast cells infected with HCMV. Previous studies have shown that tricin has anti-HCMV properties based on binding with cyclin-dependent kinase 9 (CDK9), so its mechanism of action is different from that of ganciclovir. The antiviral activity was not as strong as that of ganciclovir, but as a result of fluorination, tricin showed stronger binding to CDK9 and enhanced anti-HCMV properties. The EC_50_ of **20** was 0.126 nM, significantly better than that of ganciclovir (EC_50_ 27.5 nM) and tricin (54.3 nM). Neither **20** nor tricin showed cytotoxicity against HEL cells. In the study, 7-fluoro-3′,5′-dimethoxyflavone was also applied. It was found that the anti-HCMV effect of **20** (fluorine group at C6 position) was up to 1000 times stronger than that of tricin and the compound with fluorine at the C7 position [114]. Troshkova et al. [75] studied the properties of fluorinated flavanones against influenza virus A/Puerto Rico/8/34 (H1N1) in the MDCK cell line. They noted that the SI of the fluorinated compounds was very low, around 10 or less, and it was accompanied by high cytotoxicity towards MDCK cells (CC_50_ between 9 and 107 µM). Simultaneously, fluorinated compounds showed good antiviral properties, with IC_50_ values between 3 and 33 µM. Only flavanones with fluorine at positions C7 and C6 or C7 and C4′ exhibited weak antiviral properties. The compound, 6,8-difluoro-4′-trifluoromethylflavanone (**21**), revealed high CC_50_ (>915 µM), a selectivity index of 150, and a low IC_50_ (6 µM). For comparison, the values for oseltamivir carboxylate, the primary drug used for the treatment of influenza type A and B infections, were CC_50_ > 100 µM, IC_50_ = 0.18 µM, and SI = 556. Therefore, flavonoid derivative **21** was selected for further studies against influenza virus A/mallard/Pennsylvania/10218/84 (H5N2) and B/Florida/04/06, also in the MDCK cell line. The compound showed good but slightly weaker antiviral properties, with SI values of 53 and 42, respectively [75].

### 4.3. Anticancer Properties

Flavonoids and chalcones have shown promise in preventing and slowing the progression of various cancers, including breast, colon, liver, and lung cancers. Their anticancer effects include modulation of angiogenesis, inflammation, and oxidative stress, as well as the induction of apoptosis and cell cycle arrest. These actions are mediated through key signalling pathways, such as NF-*κ*B, and by targeting specific enzymes such as xanthine oxidase. Some of these compounds also influence non-coding RNAs involved in tumour regulation. In certain cases, flavones can act as pro-oxidants and induce ROS-mediated apoptosis in cancer cells [31,41,115].

**Figure 15:**
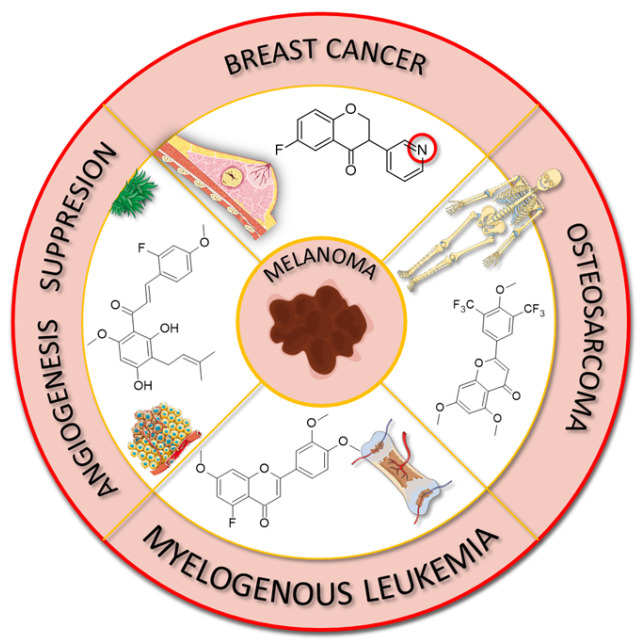
Fluorinated derivatives of flavonoids and chalcones with potential anticancer properties.

**Figure 16:**
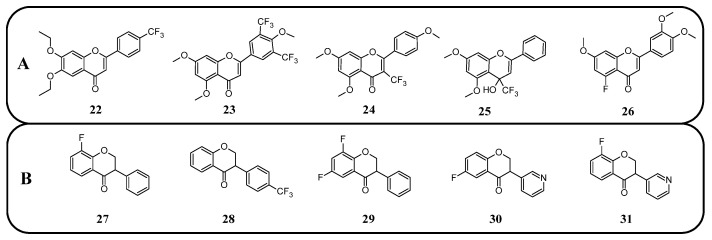
(**A**) Fluorinated flavonoid derivatives with antitumour properties (**22**–**26**). (**B**) Fluorinated isoflavanone derivatives (**27**–**31**) synthesised by Amato et al. [94].

The anticancer properties of fluorinated derivatives of flavonoids and chalcones are widely studied and tested (Figure 15, Table 4). An in vitro analysis of inhibitory activity conducted on A549 (lung) and HepG2 (liver) carcinoma cell lines showed that fluorination and trifluoromethylation at the C3′ and C4′ positions of the B-ring in chrysin and its derivatives resulted in higher IC_50_ values than unmodified chrysin, indicating weaker cytotoxicity. No toxic effect was observed against A549 cells, while IC_50_ values ranged from 33.5 to 59.4 μM against HepG2. An assay conducted on the Cellosaurus cell line (SGC-7901) revealed that trifluoromethylation at position C4′ reduced the anticancer properties of chrysin (IC_50_ increased from 5.8 to 6.6 μM). However, only after the trifluoromethylation and ethylation of both hydroxyl groups, the product 22 was obtained with superior properties (IC_50_ = 2.7 μM) (Figure 16). Methylated chrysin without a fluorine group revealed an IC_50_ of 3.7 μM, suggesting that the improved activity could be linked to increased liposolubility. However, methylated chrysin with a trifluoromethyl group at position C8 exhibited antibacterial properties similar to unmodified chrysin. In vivo assays on MCF-7 (human breast cancer), HeLa (cervical cancer), and KSP (kinesin spindle protein) cell lines using chrysin derivatives demonstrated that fluorination impaired anticancer properties of compounds [78].

Another study presented the activity of trifluoromethylated compounds against the U2OS (osteosarcoma) cell line. Modified flavones with trifluoromethyl substituents attached to the B-ring presented a stronger inhibitory effect on U2OS cells compared to other modifications of the C-ring. Notably, 3′,5′-ditrifluoromethyl-4′,5,7-trimethoxyflavone (**23**) showed the strongest inhibitory activity towards cells in the G2/M phase of the cell cycle, while the G1 and S phases were reduced to approximately 50%. Trifluoromethylation at C3 in methylated apigenin (3-trifluoromethyl-4′,5,7-trimethoxyflavone) (**24**) resulted in weaker inhibition of the G2/M phase (7%). Flavonoid derivatives with a trifluoromethyl group at the C4 position (**25**) exhibited strong cytotoxic effects, killing all U2OS cells at a concentration of just 500 nM. This suggests that the simultaneous presence of both an OH and a CF_3_ group at the C4 position leads to exceptionally high cytotoxicity. The presented compounds were not tested on healthy cells [101].

The flavonoid derivative, 5-fluoro-3′,4′,7-trimethoxyflavone (**26**), synthesised by Tsunekawa et al. [91], was tested in a highly specific context to evaluate the potential to reverse drug resistance mediated by breast cancer resistance protein (BCRP)/ATP-binding cassette subfamily G member 2. Its ability to reverse resistance to 7-ethyl-10-hydroxycamptothecin (SN-38) was investigated using human chronic myelogenous leukaemia cell lines expressing BCRP (K562/BCRP) and non-expressing control cells (K562). The concentration required to achieve a two-fold reduction in drug sensitivity (RI_50_) for **26** was determined to be 25 µM. Unfortunately, the fluorinated compound showed a weaker reversal effect compared to the compound with a hydroxyl group instead of the fluorine atom, which had a much lower RI_50_ value of 7.4 µM. This experiment demonstrates that replacing a hydroxyl group with a fluorine atom does not always result in improved properties [91].

Fluorinated isoflavanones and their derivatives obtained by Amato et al. [94] were tested for anticancer activity on oestrogen-dependent MCF-7 (human breast cancer) cell lines. The IC_50_ values of isoflavonone modified with one or two fluorine substituents ranged between 15 and 35 μM, with 8-fluoroisoflavanone **27** (Figure 16) showing the best activity (IC_50_ 15 μM) and logP values between 3 and 3.7. No clear correlation was observed between fluorine positioning in the molecule and anticancer properties. Compounds that did not exhibit anticancer activity included 4′-trifluoromethylisoflavanone (**28**) and 6,8-difluoroisoflavanone (**29**). Unlike the standard anticancer drug letrozole, the modified isoflavanones did not show significant mutagenic, tumorigenic, irritating, or reproductive toxicity in in silico tests. Only compounds with fluorination of the B-ring demonstrated a moderate influence on cell reproduction. The most potent isoflavonoid derivative, with an IC_50_ of 0.8 μM and a logP of 1.83, was 6-fluoro-3-(pyridin-3-yl)chroman-4-one (**30**). Interestingly, 8-fluoro-3-(pyridin-3-yl)chroman-4-one (**31**) exhibited the same logP value but significantly lower activity (IC_50_ 67 μM). The commercially available drug letrozole demonstrated significantly superior activity, with an IC_50_ of 0.0028 μM, despite having slightly worse lipophilicity (logP of 2.15). Fluorinated isoflavanones and their derivatives appear to be promising candidates for further investigation into reducing hormone-dependent breast cancer cell proliferation [94].

**Figure 17:**
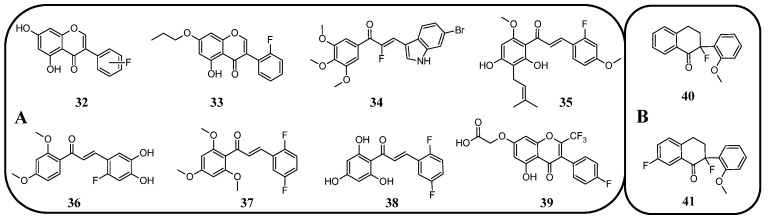
(**A**) Fluorinated isoflavonone and chalcone derivatives with antitumour properties (**32**–**39**). (**B**) Fluorinated 1-carbaisoflavanones (**40**, **41**) synthesised by Caleffi et al. [96].

Further analysis of the natural isoflavonoid genistein and its fluorinated derivatives was conducted by Yang et al. [95]. The study focused on the relationship between fluorine localisation in the B-ring and the properties of the molecule. Additionally, the hydroxyl group in position C7 was alkylated with different simple alkyl chains. The anticancer properties of the obtained genistein analogues **32** (Figure 17) were tested against three cancer cell lines: MCF-7 (breast cancer), MDA-MB-231 (triple-negative breast cancer), and MDA-MB-435 (melanoma). For comparison, genistein showed IC_50_ values ranging between 20 and 24 μM, while tamoxifen (a clinical anti-breast cancer drug) exhibited IC_50_ values of 9.13, 10.94, and 5.87 μM, respectively. The analysis revealed that the presence of a hydroxyl group increased the toxicity of the derivatives in each series of compounds. Notably, derivatives with a single fluorine atom demonstrated strong anticancer properties. For 4′-fluoro-5,7-dihydroxyisoflavone, the IC_50_ values were 6.80, 9.82, and 13.24 μM, and for 5′-fluoro-5,7-dihydroxyisoflavone, the IC_50_ values were 6.79, 13.27, and 12.22 μM, respectively. The alkylated flavonoid 2′-fluoro-5-hydroxy-7-propoxyisoflavone (**33**) exhibited similarly potent antineoplastic properties, especially against melanoma (IC_50_ 6.18 μM). The study also revealed that the elongation of the C7 alkoxy group is accompanied by a decrease in anticancer properties. However, the toxicity increased again when the chain exceeded seven carbon atoms. Moreover, no clear correlation was observed between other fluorine substitution patterns (number of fluorinated carbons: C2′, C2′ and C4′, C3′ and C4′, or C3′ and C4′) and anticancer properties [95].

A broad study of the antiproliferative properties of *α*-fluorinated chalcones was conducted by Sun et al. [116]. The compounds were tested on six cell lines: A549, HeLa, MCF-7, U937, MGC-803, and HepG2. In particular, one of the derivatives **34**, exhibited good anticancer properties, although it had very weak effects on HepG2 cells. A comparison with combretastatin A4 (4 µM) showed that the fluorinated derivative presented weaker antiproliferative action (IC_50_ values ranging from 0.025 to 0.254 µM, compared to the IC_50_ of combretastatin, which ranges from 0.011 to 0.0003 µM). However, the derivative still presented a higher selectivity ratio (11.5 vs. 2.5 for the drug). Detailed analysis revealed that the fluorinated compound induced cell cycle arrest in MGC-803 cells at the G2/M phase by regulating the expression of proteins (p-Cdc2, Cyclin B1, and p21). The induction of apoptosis in MGC-803 cells may be related to the activation of caspases-3/-7/-9. Studies on HUVECs (human umbilical vein endothelial cells) clearly demonstrated that the tested compound, even at lower doses of 2.5 μM, was capable of inhibiting tumour blood vessel formation, which results in the suppression of angiogenesis [116]. To evaluate its antiangiogenic activity, an attempt was also made to modify the fluorinated natural chalcone xanthohumol, a compound found in hops (*Humulus lupulus*). In vitro assays conducted on HUVECs with different concentrations of the compounds showed their weak activity at 5 µM. However, at 20 µM, a sudden increase in cell mortality was observed for xanthohumol with the methoxy group at the para position and fluorine at C2′ in the B-ring: (*E*)-1-(2,4-dihydroxy-6-methoxy-3-(3-methylbut-2-en-1-yl)phenyl)-3-(2-fluoro-4-methoxyphenyl)prop-2-en-1-one (**35**). This compound most effectively inhibited cell migration and adhesion. Migration assays revealed that the presence of a hydroxyl group at the C4 position significantly reduced cell motility, but this effect did not appear to depend on the fluorine position in the B-ring. Additionally, compounds containing fluorine and two MOM (methoxymethyl) groups in the A-ring exhibited strong antiproliferative and cell growth-inhibitory properties. Morphogenesis studies in vitro at 10 µM showed that, after 6 h, the xanthohumol derivative with a para-methoxy group and fluorine at C2′ again demonstrated the strongest activity. All fluorinated derivatives inhibited morphogenesis at least twice as effectively as unmodified xanthohumol [117]. An analysis of several mono- and difluorinated chalcones with two methoxy groups in the A-ring and two hydroxyl groups in the B-ring identified 2,4-dimethoxy-6′-fluoro-3′,4′-dihydroxychalcone (**36**) as the most potent antiproliferative compound. This chalcone was tested using a human cancer cell (HCC) panel consisting of 39 cell lines. The results showed strong antiproliferative activity against certain cell lines, including MCF7, HBC-4 and HBC-5 (breast), NCI-H522 (lung), OVCAR-3 (ovarian), and MKN128 (gastric), with GI_50_ values between 0.5 and 3 µM. However, some cancer types showed resistance, particularly renal, melanoma, and prostate HCC, as well as most ovarian and CNS-derived HCC lines. The most resistant was MDA-MB-231 (breast cancer), with a GI_50_ of 38 µM. A key finding was that adding a second fluorine atom at C5′ drastically reduced or eliminated antiproliferative activity [79].

Burmaoglu et al. [80] conducted further studies on approximately 70 fluorinated chalcones across HEK293, A549, A498, HeLa, A375, and HepG2 cell lines, yielding key findings. They concluded that the removal of the ketone or double bond in the chalcone linker can enhance antiproliferative activity, depending on A-ring substitutions (methoxy/hydroxyl groups). Moreover, they noted that substitution at the B-ring increases antitumour potency, especially with two fluoro groups at positions 2 and 5 against A549 and A498 cell lines. It is worth noting that HEK293 cells showed strong resistance to fluorinated chalcones. The most potent and selective compound was (*E*)-3-(2,5-difluorophenyl)-1-(2,4,6-trimethoxyphenyl)prop-2-en-1-one (**37**), with IC_50_ values ranging from 0.03 µM (A498) to 0.12 µM (A549) and an SI between 10 (A549) and 20 (A498). The highest specificity, combined with strong antitumour activity (SI 25 against HeLa and HepG2), was observed for (*E*)-3-(2,5-difluorophenyl)-1-(2,4,6-trihydroxyphenyl)prop-2-en-1-one (**38**) [80].

Fluorinated isoflavone derivatives of daidzein synthesised by Ayoup et al. [118] did not reveal improved biological activity. The IC_50_ value of daidzein against MCF-7 breast cancer cells was 11.87 μM, with an SI of 6.5. Substitution of the hydroxyl group at the 4′ position with a fluorine atom and the introduction of a trifluoromethyl group at the C2 position resulted in a derivative with reduced anticancer activity (IC_50_ 15.43 μM; SI 5.3). Additionally, it was shown that daidzein and its derivatives are only weakly active against B16F10 melanoma cells. The only compound with slightly improved anticancer properties with IC_50_ 11.23 μM and SI 6.4) was derivative **39** [118].

Other research investigating the antineoplastic activity of monofluoroisoflavonoids utilised 1-carbaisoflavanone derivatives (Figure 17). The 1-carba-3-fluoro-6′-methoxy-isoflavanone (**40**) exhibited the best anticancer properties, with the highest selectivity against MCF-7 breast cancer cells (IC_50_ 60 μM). Additionally, 1-carba-3,6-difluoro-6′-methoxy-isoflavanone (**41**) exhibited anticancer activity against chronic myeloid leukaemia cell lines, such as K562, Lucena I, and FEPS, with IC_50_ values ranging from 27 to 63 μM. These two studies highlight the superiority of fluorinated isoflavonoids over carbaisoflavonoids in the treatment of breast cancer [96].

### 4.4. Neuroprotective Properties

Flavonoids were reported to cross the blood–brain barrier (BBB). A low molecular weight, high lipophilicity, and small topological polar surface area (TPSA) are factors that promote BBB permeation of flavonoids. Therefore, naringenin and quercetin are examples of flavonoids with good and moderate permeability, respectively, while rutin and hesperidin are those with low potential, mainly because of the presence of saccharides, which increase molecular weight and TPSA and lower lipophilicity [119,120,121]. Fluorination is interesting because of the augmentation of two aspects: activity [122,123,124,125] and BBB permeation [126]. The introduction of fluorine, which is a small, electronegative atom, affects lipophilicity and electronic properties, which facilitates the interaction with the BBB [126].

**Figure 18:**
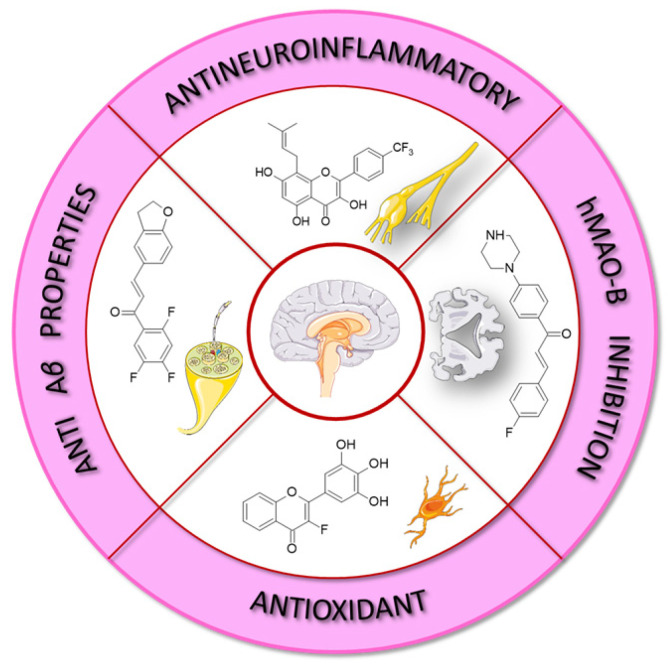
Fluorinated derivatives of flavonoids and chalcones with potential neuroprotective properties. hMAO-B—human monoamine oxidase B.

**Figure 19:**
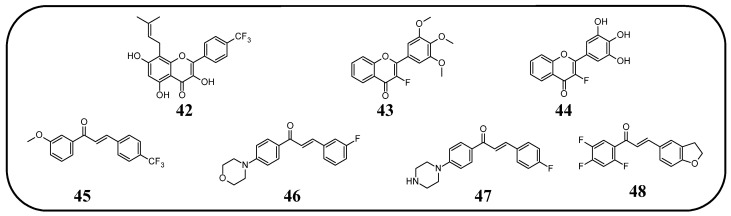
Fluorinated flavonoids and chalcones (**42**–**48**) with neuroprotective activity.

In many studies presented below, fluorinated derivatives of flavonoids and chalcones revealed potential neuroprotective properties (Figure 18). Jia et al. [122,123,124,125] performed a study on fluorinated icaritin and explored the neuroprotective properties of these compounds. Trifluoroicaritin (**42**) (Figure 19, Table 5) was evaluated in vivo using a rat model of spared nerve injury (SNI)-induced neuropathic pain. The effective dose of compound **42** was 5.0 mg/kg, and its therapeutic efficacy became evident from day 1, persisting until day 21. The movement assay (CatWalk-automated gait analysis) demonstrated that **42** increased paw pressure and improved motor coordination, indicating a reduction in pain hypersensitivity. The rotarod test confirmed an improvement in balance and motor abilities. A positive correlation was also observed between contact pressure intensity and rotational speed, suggesting that **42** influences both sensory and motor functions. Compound **42** effectively reduced mechanical allodynia (a condition where pain is caused by stimuli that normally do not cause pain) but had no effect on thermal hyperalgesia (increased sensitivity to painful heat) [122]. The results suggest that **42** exerts an analgesic effect by modulating *α*7nAChR-dependent pathways (*α*7 nicotinic acetylcholine receptors), which inhibit the BDNF/TrkB/KCC2 signalling cascade, crucial for neuroinflammatory processes and pain hypersensitivity in SNI rats. Additionally, the flavonoid increased the levels of anti-inflammatory markers (IL-10, CD206) and reduced pro-inflammatory cytokines (IL-1*β*, CD40). The optimal dose of **42** was 5 mg/kg body mass, effectively alleviating pain and anxiety symptoms [123]. Other in vivo tests conducted on rat models with complete Freund’s adjuvant (CFA)-induced chronic inflammatory pain showed the impact of **42** on microglia. Administration of the xenobiotic at doses of 3 mg/kg and 10 mg/kg equally reduced motor dysfunction and pain perception. Flavonoid derivative **42** demonstrated the ability to activate microglia, as confirmed by its capacity to increase CB2 receptor levels and decrease P2Y12 receptor levels (suppressing neuroinflammatory responses). The flavonoid further reduced the inflammatory response by influencing the expression of CD11b and iNOS, blocking the release of cytokines such as IL-1*β*, IL-6, and TNF-*α*. Finally, compound **42** promoted the transformation of microglia towards the anti-inflammatory (M2) phenotype by activating the IL-10/*β*-endorphin pathway, alleviating chronic inflammatory pain in CFA rats [125].

Alshammari et al. [90] studied the antioxidant properties of monofluoroflavones using the 1,1-diphenyl-2-picryl-hydrazyl (DPPH) antioxidant assay, which revealed better radical-scavenging activity compared to non-fluorinated flavones. The EC_50_ of 3-fluoro-3′,4′,5′-trimethoxyflavone (**43**) was 37 μg/mL and 0.24 μg/mL for 3-fluoro-3′,4′,5′-trihydroxyflavone (**44**). In comparison, the EC_50_ values for non-fluorinated counterparts were 71 μg/mL and 0.33 μg/mL, respectively. In this test, the EC_50_ value of vitamin C was determined to be 0.16 μg/mL. The neuroprotective properties of the fluoroflavones were assessed using a Sprague-Dawley rat cortical neurons in an oxidative glutamate toxicity assay. The test measures glutamate toxicity resulting from the induction of oxidative stress caused by depletion of intracellular glutathione levels. First, cells are exposed to glutamate; then, ROS are added to enhance oxidative stress, followed by assessment of cell viability. The data indicate that in vitro, C3-fluorinated hydroxyflavonoids exhibit two-fold greater neuroprotective activity than their fluorinated methoxy derivatives [90].

Fluorinated chalcones, due to their structural similarity to drugs such as fluoxetine and melperone, have become a subject of interest for neurologists. Mathew et al. [81,82] examined a series of different 5′-methoxychalcones with fluorine in the B-ring in terms of their ability to inhibit human monoamine oxidase B and A (hMAO-B and hMAO-A). hMAO-B is an enzyme potentially playing a role in the development of neurodegenerative diseases such as Alzheimer’s and Parkinson’s disease [127]. hMAO inhibitors prolong the activity of dopamine, making them preferred treatments in the early stages of Parkinson’s disease (e.g., selegiline). The fluorinated chalcones showed very weak binding to hMAO-A but significantly better affinity for hMAO-B. The most active compounds were those with fluorinated groups at position C4, particularly the compound with a trifluoromethyl group (**45**). Along with the activity, there was a high selectivity index between the two enzymes (hMAO-B/hMAO-A equals 0.05 for the chalcone and 0.04 for selegiline). Reversibility tests showed, however, that chalcones bind weakly to the enzyme. Of the initial 87% hMAO-B inhibition, only 11% remained after washing, whereas for selegiline, the value remained stable at around 47% [82]. In subsequent studies, fluorinated chalcones were tested with morpholine or imidazole at the C4′ position. They were examined in the context of their ability to inhibit hMAO-A, hMAO-B, and acetylcholinesterase (AChE). Among the morpholine derivatives, the compound with fluorine at the C3 position—(2*E*)-3-(3-fluorophenyl)-1-[4-(morpholin-4-yl)-phenyl]prop-2-en-1-one (**46**)—demonstrated the best hMAO-B inhibitory activity, comparable to commercial drugs (IC_50_ 0.087 µM, which is better than pargyline). The selectivity index of this compound was very high (hMAO-A/hMAO-B—512). This compound was found to be a reversible inhibitor in further studies. The compound with fluorine at the C2 position exhibited slightly weaker properties. The presence of fluorine or a trifluoromethyl group at the C4 position resulted in reduced MAO-B inhibitory activity. However, fluorinated morpholine-containing derivatives still demonstrated significantly better properties than imidazole-containing derivatives. The chalcones showed no affinity for AChE. Additionally, the parallel artificial membrane permeation assay (PAMPA) was conducted to determine the blood–brain barrier (BBB) permeation potential of the morpholine-containing chalcones. All derivatives exhibited permeability similar to that of testosterone (approximately 19 × 10⁻⁶ cm/s) [83].

An attempt was also made to introduce a piperazine moiety at the C3 position. Compounds containing fluorine (**47**) or trifluoromethyl group at the C4′ position exhibited the lowest IC_50_ values against hMAO-B among all tested compounds (0.65 and 0.71 µM). These were the only compounds that showed activity against butyrylcholinesterase- BChE (approximately 36 µM) and AChE (approximately 27 µM). Unfortunately, their selectivity index—around 49, was lower than that of the morpholine derivatives. Piperazine derivatives also proved to be reversible inhibitors. Additionally, fluorinated compounds demonstrated the best properties among the halogenated derivatives [84].

Sánchez et al. [128] examined the neuroprotective properties of chalcones with a halogenated A-ring and a benzodihydrofuran moiety instead of the classical C-ring. Tests were conducted on the PC-12 cell line, derived from a rat adrenal medulla pheochromocytoma. The cells were incubated for 24 h at various concentrations, with a separate assessment of cells treated with amyloid *β* peptide (A*β*). Once again, fluorinated compounds outperformed other halogen derivatives. Notably, the compound with fluorine atoms at positions C2, C4, and C6- (*E*)-3-(2,3-dihydrobenzofuran-5-yl)-1-(2,4,5-trifluorophenyl)prop-2-en-1-one (**48**) exhibited low cytotoxicity and provided up to 150% cell viability in A*β*-treated cells, whereas without this compound, cell survival was approximately 70% [128].

**Table 5:** Short summary of the neuroprotective properties of fluorinated derivatives of flavonoids and chalcones.

To Sum Up
Pros
Fluorinated flavonoids exhibit neuroprotective properties through a complex mechanism. They act as antioxidants, suppress neuroinflammation, inhibit the activity of MAO-B enzymes, and protect cells from the effects of amyloid *β* peptide [128]. Fluorinated chalcones modified with a morpholine group are capable of good blood–brain barrier permeation [83,84].
**Cons**
Fluorinated chalcones present only weak affinities for cholinesterases [84].

### 4.5. Other Properties

The remaining studies describe the potential anti-inflammatory and antioxidant properties of fluorinated derivatives of flavonoids and chalcones, as well as their applications in diagnostics. Flavonoids are known to exhibit anti-inflammatory activities via different mechanisms, especially by the inhibition of the synthesis and actions of specific pro-inflammatory mediators such as eicosanoids, cytokines, adhesion molecules, and C-reactive protein, as well as transcription factors and regulatory enzymes. It is important that flavonoids can inhibit the onset and development of inflammatory diseases [17,18,19,20]. These molecules can downregulate mast cell activation, which is responsible for secreting inflammatory mediators such as histamine and pro-inflammatory cytokines [18]. Flavonoids as polyphenols present high antioxidant activity and can scavenge free radicals, donate hydrogen atoms, and chelate metal cations. The main antioxidant mechanism of action relies on transferring hydrogen atoms to free radicals [12]. These natural products, as potent antioxidants, reveal the potential to attenuate tissue damage or fibrosis [17].

**Figure 20:**
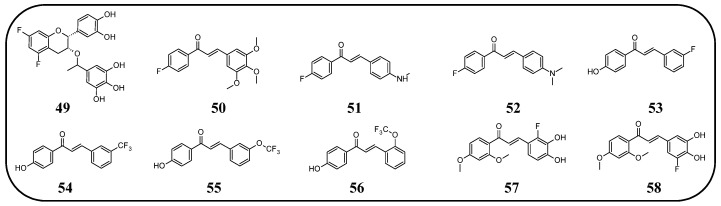
Other fluorinated flavonoids and chalcones (**49**–**58**).

Below, the anti-inflammatory and antioxidant properties of fluorinated derivatives of flavonoids and chalcones, as well as their applications in diagnostics, are briefly discussed and summarised (Table 6). Stadlbauer et al. [93] highlighted the anti-inflammatory properties of polyphenols found in green tea. The activity of 3-*O*-gallates, specifically epicatechin-3-*O*-gallate (ECG), the second most prevalent polyphenol in green tea, and its fluorinated derivative (−)-5,7-difluoro-epicatechin-3-*O*-gallate **49** (Figure 20), was investigated. Both compounds contain the flavanol catechin. Assays demonstrated that **49** reduced the viability of human lymphocyte cells. The proliferation of activated human lymphocytes was inhibited to almost 0% at a concentration of 30 µM, whereas ECG achieved a similar effect only at 100 µM. However, the reduction in cell proliferation was due to the induction of apoptosis. The antiproliferative properties of **49** indicate that it could be used to treat chronic inflammation [93].

Fluorinated chalcones were also examined for their anti-inflammatory potential, particularly their ability to inhibit nitric oxide production. NO plays a crucial role as a vasodilator, contributing to oedema formation, leukocyte activation, and cytokine production. Additionally, its reaction with superoxide anions leads to the formation of peroxynitrite, a compound known to cause tissue damage. In the study by Rojas et al. [129], the A-ring of the chalcones was modified with one or two fluorine atoms or a trifluoromethyl moiety, while the B-ring contained either two or three methoxy groups. The results indicated that trimethoxychalcone derivatives with a fluorine atom at the C4 position were significantly more effective at inhibiting NO production compared to those containing a trifluoromethyl group. Interestingly, the trifluoromethyl moiety provided good inhibition when positioned at C2, regardless of the number or positioning of methoxy groups in the B-ring. The IC_50_ values of the tested compounds ranged from 0.28 to 0.91 µM, with monofluorinated derivatives consistently exhibiting stronger NO inhibition than their trifluoromethyl analogues. However, chalcones containing two fluorine atoms, including one at C2, showed slightly weaker activity compared to their monofluorinated counterparts. The most potent compound in limiting NO production was 4-fluoro-3′,4′,5′-trimethoxychalcone (**50**), which demonstrated an IC_50_ of 0.03 µM and achieved 94.1% inhibition of nitrite formation. Moreover, findings suggest that the inhibition of NO production by these fluorinated chalcones in macrophages may occur at the enzyme expression level, further highlighting their potential as anti-inflammatory agents [129].

Flavonoids and chalcones are well known for their antioxidant properties. Bist et al. [88] analysed nearly 40 fluorinated chalcone derivatives for their inhibitory activity on ROS production stimulated by lipopolysaccharide in RAW 264.7 macrophages. The analysis showed that a hydroxyl group at the *para* position in the A-ring, along with a fluorinated moiety at the *meta* position in the B-ring, are essential for inhibiting ROS production in macrophages. The IC_50_ values for ROS inhibition were lowest for the trifluoromethyl moiety (1.44 µM) (53), higher for the fluorine group (9.15 µM) (54), and the weakest activity was observed for the trifluoromethoxy group (>10 µM) (55). Chalcones containing the trifluoromethoxy group exhibited strong activity when positioned at the *ortho* position. Among these compounds, the highest activity was observed for chalcones with a hydroxyl group at C4 (IC_50_ 1.34 µM) (56), followed by C3 (IC_50_ 1.61 µM). However, a hydroxyl group at the *ortho* position did not inhibit ROS production. Additionally, the presence of a hydroxyl group at the *ortho* position of the A-ring, combined with a fluorinated B-ring, was often associated with macrophage toxicity. It was also observed that B-ring-modified chalcones with fluorine-containing groups at the C3 position exhibited no significant ROS inhibition when lacking a hydroxyl group [88].

In another study, several chalcones with two methoxy groups in the A-ring and two hydroxyl groups in the B-ring, modified with one or two fluorine atoms in the B-ring, were tested. Their activity was evaluated against Fe^3+^- ADP-induced NADPH-dependent lipid peroxidation in rat liver microsomes. The results indicated that fluorine substitution at C2′ (**57**) and C5′ (**58**), with a methoxy group at C4 and C2, provided the strongest antioxidant properties. Additionally, an analysis conducted on rat basophilic leukemia-1 (RBL-1) cells assessed the inhibitory action on 5-lipoxygenase. Compound **56** exhibited the highest ability to inhibit the enzyme, with an IC_50_ value of 0.012 µM. This value was the same for the analogue with fluorine at position C6′ or at both C6′ and C5′ (with methoxy moieties at C2 and C5) [79].

A fluorine group has also been applied as a labelling agent in diagnostics. The ^18^F isotope is widely used in positron emission tomography (PET) imaging (e.g., in the structure of fluorodeoxyglucose-FDG). An attempt was made to introduce this isotope into the chalcone structure, resulting in (*E*)-3-(4-(methylamino)phenyl)-1-(4-fluorophenyl)-2-propen-1-one (**51**) and (*E*)-3-(4-(dimethylamino)phenyl)-1-(4-fluorophenyl)-2-propen-1-one (**52**). First, in vivo biodistribution studies in healthy mice showed that the brain uptake of the radiopharmaceuticals after 2 min was similar for **51** (5.47% ID/g brain, injected dose per gram of brain) and florbetapir, a commonly used radiopharmaceutical (4.90% ID/g brain). For **52**, the uptake was 4.43% ID/g brain. However, after 30 min, the retention of both fluorinated chalcones was nearly three times lower compared to the commercial radiopharmaceutical. The in vitro studies focused on the ability of the compounds to bind *β*-amyloid (A*β*) plaques. The dissociation constant (K_d_) values ranged from 4.5 to 6.5 nM, indicating a high affinity for A*β*_1–42_ aggregates. For comparison, [^3^H]PiB has a K_d_ 4.7 nM. Subsequently, in vitro autoradiography (ARG) was performed using human brain sections with Alzheimer’s disease pathology. The compounds with intensely labelled A*β* plaques and did not accumulate in regions without A*β* plaques. The conclusions drawn from these experiments suggest that **52** and **51** may be useful in the PET imaging of A*β* plaques in the diseased brain. However, one of the major concerns regarding the use of these compounds is their rapid clearance from the body [87].

**Table 6:** Short summary of the anti-inflammatory and antioxidant properties of fluorinated derivatives of flavonoids and chalcones, as well as their applications in diagnostics.

To Sum Up
Pros
Fluorinated flavonoids exhibit anti-inflammatory properties by reducing lymphocyte viability and inhibiting nitric oxide production [93,129]. Due to the ability of fluorinated chalcone derivatives to cross the BBB and their strong binding to *β*-amyloid (A*β*) plaques, they are promising candidates for neurodegenerative disease imaging [87]. Fluorination of hydroxyl-containing flavonoids enhances their antioxidant properties [88].
**Cons**
A significant drawback in using chalcones for imaging is their rapid elimination from the body [87].

## 5. Conclusions

Flavonoids and chalcones constitute a widely recognised group of compounds that have garnered attention due to their diverse biological activities and potential therapeutic applications.

The synthesis of fluorinated flavonoids and chalcones based on various methods was presented, including condensation and cyclisation reactions starting from fluorinated precursors, as well as fluorination strategies, including the use of molecular fluorine or fluorinating agents. The most common synthetic approach for chalcone synthesis involves the Claisen–Schmidt aldol condensation reaction and related methods (e.g., using acid chlorides). Subsequent cyclisation reactions, using catalysts, such as FeCl_3_, NFSI, or I_2_, allow for the transformation of chalcones into flavonoids, with the preservation or rearrangement of fluorine-containing rings. A more challenging method involves the direct fluorination of the flavonoid structure using agents such as NFSI, Togni I, or even molecular fluorine.

**Figure 21:**
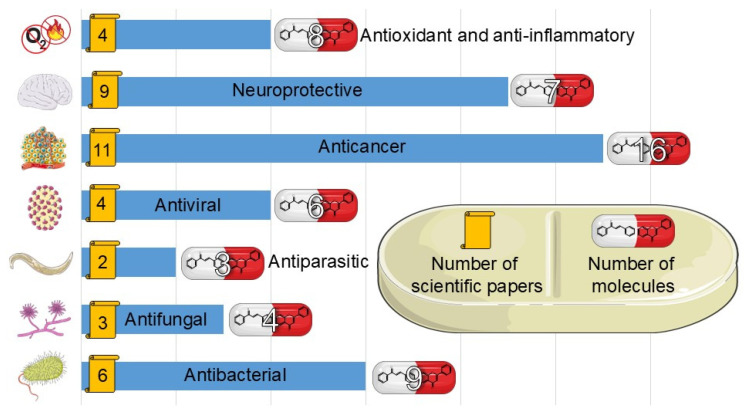
Number of scientific articles (scroll) presenting studies on the pharmacological activities of fluorinated flavonoids and chalcones, with indication of compounds possessing the highest potential (red-white pill).

This review also presents and discusses flavonoids and chalcones regarding their prospective anti-inflammatory, antidiabetic, anticancer, antiosteoporotic, cardioprotective, neuroprotective, hepatoprotective, antimicrobial, and antiparasitic applications. It is worth noting that fluorinated flavonoids and chalcones in many cases exhibited improved biological properties compared to their non-fluorinated counterparts, particularly in anticancer, antiviral, and antioxidant activities (Figure 21). The introduction of fluorine atoms into the molecular structure of flavonoids enhances lipophilicity, bioavailability, and efficacy, although it may also lead to increased cytotoxicity. It has been found that certain fluorinated flavonoids can act as efflux pump inhibitors (e.g., 3′,4′-difluoroquercetin and 8-(4-fluoroanilino) derivatives of quercetin and luteolin), increasing the effectiveness of certain antibiotics. However, some bacteria have also shown the ability to remove fluorine atoms from flavonoids, which may affect their activity. Additionally, fluorinated compounds have demonstrated antiparasitic properties against *Plasmodium* and *Leishmania* species. Interestingly, fluorinated flavonoids exhibit neuroprotective properties, as they can prevent cells from oxidative stress (e.g., 3-fluoro-3′,4′,5′-trihydroxyflavone). Fluorinated chalcones bind to monoamine oxidase B (MAO-B), especially chalcones modified with a piperazine group, which show the highest affinity, potentially influencing neurotransmitter metabolism. Additionally, fluorinated chalcones containing a benzodihydrofuran moiety prevent amyloid-*β* peptide (A*β*)-induced neurotoxicity. Both phenomena are key factors in the development of Alzheimer’s disease. Furthermore, they can modulate acetylcholine receptors, reducing neuroinflammation. They can potentially be used in the therapy of damaged nerve tissue (e.g., trifluoroicaritin). Some studies have also shown that these compounds can reduce other pro-inflammatory factors, such as nitric oxide production (e.g., 4-fluoro-3′,4′,5′-trimethoxy-chalcone) and excessive lymphocyte production (e.g., fluorinated epicatechin).

Based on the presented data, the following conclusions regarding the structure-activity relationship for fluorinated flavonoids can be formulated:

- Fluorinated compounds with hydroxyl groups demonstrate better protective effects on healthy cells and more potent anticancer properties than derivatives without these groups or those with methylated hydroxyls. This is likely due to the ability of hydroxyl groups to enhance hydrogen bonding with biological targets.
- A higher number of fluorine atoms in a molecule does not necessarily correlate with increased toxicity, challenging the assumption that polyfluorination always leads to adverse effects. Monofluorinated chalcones, for example, often outperform trifluorinated ones in antioxidant activity.
- Chalcones with a fluorine atom at the C2′ position exhibit the most potent anticancer properties.
- Monofluorinated chalcones show better antioxidant activity than their trifluorinated counterparts.
- Flavonoids with fluorine in the A-ring display superior antifungal properties compared to compounds with fluorine in the B-ring.
- The attachment of a fluorinated group at the C4 position of a flavonoid, along with the simultaneous reduction of the carbonyl group to a hydroxyl group, results in a compound with strong cytotoxic properties.
- Compounds with a fluorinated moiety in the *para* position exhibit good neuroprotective properties, likely due to enhanced interactions with neural receptors and enzymes.

Despite promising results, the practical application of fluorinated flavonoids requires further optimisation and evaluation of their properties. For example, fluorination can enhance cellular uptake, but at the same time, it may also increase cytotoxicity, particularly in compounds containing multiple fluorine atoms or specific substitution patterns. Another challenge is bioavailability. Although fluorination improves lipophilicity, the poor aqueous solubility of some derivatives can limit their systemic absorption. Novel delivery systems, such as nanoparticles or liposomes, could help overcome this limitation. Additionally, certain microorganisms can cleave fluorine atoms from flavonoids, potentially reducing their biological activity. This phenomenon requires thorough investigation and may necessitate the development of fluorinated analogues resistant to microbial metabolism.

Fluorinated flavonoids and chalcones represent a frontier in pharmaceutical research, offering enhanced biological activities, diverse therapeutic applications, and the potential to overcome drug resistance. However, further studies are necessary to adjust their pharmacological profiles. By bridging the gap between preclinical promise and clinical reality, fluorinated flavonoids and chalcones could significantly impact modern medicine, offering novel solutions to global health challenges. Further research should focus on mechanistic studies, pharmacokinetic profiling, and clinical application to completely exploit the potential of these compounds.

## Figures and Tables

**Table 2:** Short summary of the antifungal and antiparasitic properties of fluorinated derivatives of flavonoids and chalcones.

To Sum Up
Pros
Fluorinated chalcones exhibit broad antifungal properties, whereas flavonoids act only on specific species with equal effectiveness [89]. Fluorinated chalcones are particularly effective against *S. cerevisiae* [77]. Chalcones demonstrate strong antiparasitic properties against *Plasmodium* and *Leishmania* [85,86].
**Cons**
Only one of the studied compounds (**9**) was found to be effective against *Candida albicans*, with an MIC of 7.8 µM [110].

**Table 3:** Short summary of the antiviral properties of fluorinated derivatives of flavonoids and chalcones.

To Sum Up
Pros
Fluorinated chalcones exhibit interesting antiviral activity, comparable or better to some drugs in in vitro studies [75,89]. Fluorinated flavonoids exhibit low selectivity; however, this is often accompanied by their very weak antiviral properties [89]. Certain fluorinated chalcones and flavonoids appear to be effective against H1N1, H5N2, HRV 1B and 14, HCMV, and influenza B [75,76,89,114]. 6-Fluorotricin demonstrated an exceptionally high EC_50_ against HCMV (0.126 nM) while showing no cytotoxicity [114].
**Cons**
Both fluorinated chalcones and flavonoids present low selectivity indices compared to other antiviral drugs [89]. Despite the high antiviral activity of fluorinated chalcones and flavonoids, there is still a need for further structural optimisation, as these compounds reveal toxicity towards mammalian cells, which prevents their further clinical use.

**Table 4:** Short summary of the anticancer properties of fluorinated derivatives of flavonoids and chalcones.

To Sum Up
Pros
Fluorinated isoflavonoids appear to be particularly effective against breast cancer cells [94]. The fluorinated compounds inhibit U2OS (osteosarcoma) and MGC-803 (Cellosaurus) cell line growth at the G2/M phase [101,116]. Chalcones are capable of inhibiting tumour blood vessel formation, thereby blocking angiogenesis [116].
**Cons**
Fluorination combined with the removal of hydroxyl groups seems to weaken the antitumour effect of other drugs, acting in an anti-synergistic manner [95]. HEK293 cell lines appear to be resistant to fluorinated compounds [80].

## Data Availability

No new data were created or analysed in this study. Data sharing is not applicable to this article.

## Abbreviations

A*β* peptide	Amyloid *β* peptide
AChE	Acetylcholinesterase
ADP	Adenosine diphosphate
B[a]P	Benzo[a]pyrene
BBB	Blood–brain barrier
BChE	Butyrylcholinesterase
BMP	Bone morphogenetic proteins
BCRP	Breast cancer resistance protein
CC_50_	50% cytotoxic concentration
CCCP	Carbonyl cyanide 3-chlorophenylhydrazone
CDK9	Cyclin-dependent kinase 9
CFA	Complete Freund’s adjuvant
CRP	C-reactive protein
COVID	Coronavirus disease
DCM	Dichloromethane
DMA	Dimethylacetamide
DMF	Dimethylformamide
EC_50_	Half maximal effective concentration
ECG	Epicatechin-3-*O*-gallate
HCC	Human Cancer Cells
HCMV	Human cytomegalovirus
HIV	Human immunodeficiency virus
HRV	Human rhinovirus
HUVECs	Human umbilical vein endothelial cells
IC_50_	Half maximal inhibitory concentration
IL	Interleukin
iNOS	Inducible nitric oxide synthase
logP	Partition coefficient (octanol/water)
MDCK	Madin–Darby canine kidney
MIC	Minimum inhibitory concentration
MOM	Methoxymethyl group
MPO	Myeloperoxidase
MRSA	Methicillin-resistant *Staphylococcus aureus*
NADPH	Nicotinamide adenine dinucleotide phosphate (reduced form)
NF-*κ*B	Nuclear factor kappa-light-chain-enhancer of activated B cells
NFSI	N-Fluorobenzenesulfonimide
PET	Positron emission tomography
ROS	Reactive oxygen species
SARS	Severe acute respiratory syndrome
SNI	Spared nerve injury
THF	Tetrahydrofuran
TMS	Tetramethylsilane
TMSCF_3_	Trifluoromethyltrimethylsilane
TNF-α	Tumour necrosis factor alpha
